# *klf2a* ^sh317^ Mutant Zebrafish Do Not Recapitulate Morpholino-Induced Vascular and Haematopoietic Phenotypes

**DOI:** 10.1371/journal.pone.0141611

**Published:** 2015-10-27

**Authors:** Peter Novodvorsky, Oliver Watson, Caroline Gray, Robert N. Wilkinson, Scott Reeve, Carl Smythe, Richard Beniston, Karen Plant, Richard Maguire, Alexander M. K. Rothman, Stone Elworthy, Fredericus J. M. van Eeden, Timothy J. A. Chico

**Affiliations:** 1 The Bateson Centre, University of Sheffield, Sheffield, United Kingdom; 2 Department of Cardiovascular Science, University of Sheffield, Sheffield, United Kingdom; 3 Department of Biomedical Science, University of Sheffield, Sheffield, United Kingdom; Medical College of Wisconsin, UNITED STATES

## Abstract

**Introduction and Objectives:**

The zinc-finger transcription factor Krϋppel-like factor 2 (KLF2) transduces blood flow into molecular signals responsible for a wide range of responses within the vasculature. KLF2 maintains a healthy, quiescent endothelial phenotype. Previous studies report a range of phenotypes following morpholino antisense oligonucleotide-induced *klf2a* knockdown in zebrafish. Targeted genome editing is an increasingly applied method for functional assessment of candidate genes. We therefore generated a stable *klf2a* mutant zebrafish and characterised its cardiovascular and haematopoietic development.

**Methods and Results:**

Using Transcription Activator-Like Effector Nucleases (TALEN) we generated a *klf2a* mutant (*klf2a* ^sh317^) with a 14bp deletion leading to a premature stop codon in exon 2. Western blotting confirmed loss of wild type Klf2a protein and the presence of a truncated protein in *klf2a* ^sh317^ mutants. Homozygous *klf2a* ^sh317^ mutants exhibit no defects in vascular patterning, survive to adulthood and are fertile, without displaying previously described morphant phenotypes such as high-output cardiac failure, reduced haematopoetic stem cell (HSC) development or impaired formation of the 5^th^ accessory aortic arch. Homozygous *klf2a* ^sh317^ mutation did not reduce angiogenesis in zebrafish with homozygous mutations in von Hippel Lindau (*vhl*), a form of angiogenesis that is dependent on blood flow. We examined expression of three klf family members in wildtype and *klf2a* ^sh317^ zebrafish. We detected vascular expression of *klf2b* (but not *klf4a* or *biklf/klf4b/klf17*) in wildtypes but found no differences in expression that might account for the lack of phenotype in *klf2a* ^sh317^ mutants. *klf2b* morpholino knockdown did not affect heart rate or impair formation of the 5^th^ accessory aortic arch in either wildtypes or *klf2a* ^sh317^ mutants.

**Conclusions:**

The *klf2a* ^sh317^ mutation produces a truncated Klf2a protein but, unlike morpholino induced *klf2a* knockdown, does not affect cardiovascular development.

## Introduction

KLF2 belongs to the KLF zinc finger transcription factor family, characterised by three tandem C_2_H_2_ zinc fingers that serve as a DNA binding motif [1]. Since its characterisation in mice [1] and humans [2] it is clear that mammalian KLF2 plays important roles in vascular development and homeostasis [3]. KLF2 modulates expression of genes critical in regulating vascular tone [4, 5], thrombosis [6], inflammation [3, 4] barrier function [7] and antioxidative properties of endothelium [8]. Within the vascular wall, *KLF2* is expressed exclusively in endothelium [3, 5, 9]. Endothelial *KLF2* expression is induced by blood flow via mechanotransduction [3, 10], as well as via non flow-dependent mechanisms such as inflammatory cytokines or statins [4, 6, 11]. Homozygous *Klf2* knockout mice die between E12.5–14.5 from intra-embryonic and intra-amniotic haemorrhaging associated with defective blood vessel morphology. Impaired vascular smooth muscle cell and pericyte migration resulting in failure to organize into a compact tunica media has been reported [12, 13], although while another group confirmed the stage of lethality (E11.5–13.5) and abdominal bleeding, they but did not detect vessel wall abnormalities [14]. Conditional *Klf2* deletion in murine embryonic tissues confirms that endothelial *Klf2* deletion is responsible for embryonic mortality [15].

The zebrafish (*Danio rerio*) is an increasingly popular model to study vascular development [16]. Zebrafish possess two *KLF2* paralogs, *klf2a* and *klf2b* [17] and *klf2a* expression in embryonic zebrafish vasculature is (like mammalian *KLF2*) regulated by blood flow [3].

Assessment of gene function in zebrafish has often used morpholino antisense oligonucleotide-mediated gene knockdown, and several studies have reported the effect of *klf2a* knockdown on zebrafish cardiovascular and haematopoietic development. *klf2a* knockdown has been reported to cause high-output cardiac failure with pericardial oedema and high aortic blood flow velocity attributed to loss of smooth muscle tone [15]. *klf2a* knockdown induced failure to form the 5^th^ accessory aortic arch (AA5x) although with otherwise preserved vascular patterning [18]. In relation to haematopoietic development *klf2a* knockdown impaired maintenance of haematopoietic stem cell (HSC) gene expression via a blood flow dependent signalling cascade involving nitric oxide (NO) [19].

Morpholino oligonucleotides (MO) remain commonly used in zebrafish reverse genetic studies. However, their reliability has been called into question [20]. The advantages of MOs in terms of cost and speed are counterbalanced by their temporary and incomplete action and potential for off-target effects and toxicity [21]. The development of targeted genome editing to create stable, heritable mutations in genes of interest avoids the disadvantages of morpholino use. Several techniques are available, including Zinc Finger Nucleases (ZFN) [22], Transcription Activator-like Effector Nucleases (TALEN) [23] and most recently Clustered Regularly Interspaced Short Palindromic Repeats/CRISPR-associated systems (CRISPR/Cas9) [24]. These induce site-specific double stranded DNA breaks that are repaired by error-prone non-homologous end joining DNA repair resulting in mutations that can disrupt the reading frame.

Targeted mutation has been proposed to be a more reliable way of assessing gene function [20]. Recent publications have shown that several published morphants phenotypes are not reproduced in mutants [25]. Very recently, Rossi *et al*. found that the severe vascular defect induced by *egfl7* morpholino knockdown was not seen in *egfl7* mutants, due to a compensatory upregulation of Emilin 2 and 3 not seen in morphants [26].

The existence of mechanisms that compensate for the effects of loss-of-function mutations would explain the low rate of overt phenotypes seen in the Sanger zebrafish mutagenesis project [27]. However, as zebrafish were first established as a model for forward mutagenesis screens [28–30], clearly not all mutations can be so “rescued”. There is therefore a pressing need to rapidly establish which mutations can be compensated for, and which cannot.

We used TALENs to generate a stable *klf2a* mutant line (named *klf2a* ^sh317^). This mutation produces an aberrant mRNA transcript that is predicted to produce a significantly truncated klf2a protein without the essential DNA binding motifs. However, homozygous *klf2a* ^sh317^ mutants exhibit no phenotypic differences compared to wildtypes (WT) and did not reproduce previously described *klf2a* morphant phenotypes, nor did they show any effect in miR-126 levels. We examined the expression patterns of three evolutionary closest *klf* family members; *klf2b*, *klf4a* and *biklf/klf4b/klf17* in the search for a gene that might compensate for the mutation in *klf2a*. Although we detected *klf2b* expression in the vasculature, which has not been described previously, we found no differences in expression of these genes between *klf2a* ^sh317^ mutants and wildtypes, and klf2b knockdown did not reveal any phenotype in *klf2a* ^sh317^ mutants.

## Materials and Methods

### Zebrafish Husbandry

Zebrafish were raised in the Bateson Centre aquaria and fed *Artemia nauplii*. Zebrafish were kept on a constant 14 hour on /10 hour off light cycle at 28°C. Studies performed on zebrafish conformed to Directive 2010/63/EU of the European Parliament and Home Office regulations under Home Office project licences 40/3434 held by TJAC and 40/3082 held by FVE.

### Fish Strains

*hey2*/*gridlock* mutants [31] were kindly supplied by Randall Peterson. *vhl* ^hu2117^ [32] was obtained from Hubrecht Institute in Utrecht, Netherlands and was crossed with *Tg(fli1*:*eGFP)* [33] kindly supplied by Brant Weinstein. *Tg(kdrl*:*HRAS-mCherry)* [34] was kindly supplied by Arndt Siekmann and *Tg(flk1*:*EGFP-nls)* was kindly supplied by Markus Affolter. *klf2a* ^sh317^ mutant line was generated in Nacre wild type (WT) strain and was crossed with *Tg(kdrl*:*HRAS-mCherry;flk1*:*EGFP-nls*) and with *vhl* ^hu2117 +^/^−^;*Tg(fli1*:*eGFP)*.

### Morpholino Studies

Morpholino oligonucleotides (GENE-TOOLS, OR, USA) were diluted with ultra-pure (miliQ) water and phenol red (SIGMA) and 2ng of each were injected into one-cell stage embryos. Morpholino sequences: *tnnt2* morpholino 5′ CATGTTTGCTCTGACTTGACACGCA 3′ as published [35], *klf2b* morpholino 5′ AGTGTCAAATACTTACATCCTCCCA 3′, control morpholino 5′ CCTCTTACCTCAGTTACAATTTATA 3′ (GENE TOOLS stock control).

### Drug Treatments

In order to reversibly block cardiac contraction and stop blood flow embryos were treated with the anaesthetic tricaine [2.53mM] or the myosin ATPase inhibitor 2,3-butanedione 2-monoxime (BDM) [15mM] (both SIGMA) from 46hpf until 65hpf as previously published [18, 36].

## RNA Extraction, Reverse Transcription and Polymerase Chain Reaction

Total RNA was extracted from pools of 30 embryos or unfertilised eggs using TRIzol (LIFE TECHNOLOGIES) according to manufacturer`s instructions. Total RNA was reversely transcribed into complementary DNA using the VERSO cDNA Synthesis Kit (THERMO SCIENTIFIC). Primers for amplification of *klf2a*, *klf2b*, and *gapdh* were: *klf2a* F 5`GGATCCATGGCTTTGAGTGGAACG 3`, *klf2a* R 5`GAATTCCTACATATGACGTTTCAT 3`, *klf2b* F 5'-GCCTTCGATTTCAACGTTTGC-3', *klf2b* R 5'-ATCCTCGTCATCCTTACGGC-3', *gapdh* F 5`AGGCTTCTCACAAACGAGGA, *gapdh* R 5`GCCATCAGGTCACATACACG 3`.

Quantitative PCR (qPCR) of zebrafish mature miR-126-5p was performed according to the manufacturer’s instructions using the TaqMan MicroRNA Assay. Total RNA from 72hpf zebrafish embryos was purified using Trizol. cDNA was prepared using the TaqMan microRNA Reverse Transcription kit (4366596, APPLIED BIOSYSTEMS) and qPCR performed using miR-126-5p and U6 TaqMan Assays (4427975 and 4427975, APPLIED BIOSYSTEMS). The primers used for reverse transcription and qPCR were supplied with each assay. The qPCR primer is a stem-loop oligonucleotide containing the sequence of the mature miR-126-5p (CAUUAUUACUUUUGGUACGCG). qPCR was performed in duplicate using an ABI 7900HT Fast Real-Time PCR System. Expression of miR-126-5p was normalized to U6 for relative quantification. Individual points represent relative quantification of miR-126-5p in a pool of embryos relative to mean expression in the wild-type group. Summary data is displayed as mean ± standard error of the mean (SEM). Statistical comparison was made using a two-tailed Student’s *t*-test on log-transformed data.

### Measurement of Cardiovascular Performance

#### Heart Rate

Embryos were kept in individual dishes and individually removed from the 28°C incubator immediately prior to measurement to minimize the effect of environmental temperature on heart rate [37]. Heart rates were measured directly by eye under a stereomicroscope for 30 seconds.

#### Blood flow velocity

Particle image velocimetry (PIV) analysis using erythrocytes as tracer particles was used to measure aortic blood velocity in the mid aorta immediately above the cloaca. Zebrafish embryos were imaged at 10x magnification and at 300 frames/second using a high speed camera (OLYMPUS IX81) and Video Savant 4.0 digital video recording software (IO INDUSTRIES). 400 images (frames) in.tiff format per each embryo were recorded. Images were analysed using ImageJ (version 1.45s public domain software http://imagej.nih.gov/ij) to obtain a kymograph with lines representing movements of individual erythrocytes in real time. Kymographs were uploaded to custom made software CORRELATOR which calculated instantaneous erythrocyte velocities throughout the cardiac cycle. Knowing the ratios of pixel/μm (0.8pixel/μm), pixel/frame (1:1) and the rate of imaging (300frames/second), velocity (v) was calculated as follows: v = distance/time [μm/sec]. Average erythrocyte velocity per cardiac cycle was calculated by averaging all instantaneous velocities measured during the cardiac cycle. Additionally, cardiac cycles from all embryos of the same group were put in phase, and average instantaneous erythrocyte velocities for each frame were calculated and plotted as a single velocity curve.

### Whole-Mount In Situ Hybridization (WISH)

WISH was performed according to previously published protocols [38, 39]. Embryos were permeabilized with proteinase K (ROCHE) [15μg/ml] for following times depending on the developmental stage: 0min (24hpf), 15min (36hpf), 40min (48hpf), 80min (72hpf), 100min (4dpf) and 150min (5dpf). Maleic acid buffer was used instead of phosphate buffered saline for day 2 and day 3 washes. Expression was detected with digoxigenin (ROCHE) labelled riboprobes and resolved using BM Purple (ROCHE). EST clone IMAGp998A0510285Q (IMAGENES) containing full-length *klf2a* cDNA sequence was digested with XBaI and EcoRI (both NEB) resulting in a 1144bp fragment which was inserted into Bluescript KS expression plasmid and subsequently used for *klf2a* antisense riboprobe synthesis (EcoRI (NEB) and T7 RNA polymerase (ROCHE)). Total embryonic RNA at 48hpf was used as a template for *klf2b*, *klf4a* and *biklf/klf4b/klf17* riboprobes synthesis. The following primers were designed to amplify approx. 1000bp cDNA fragments: *klf2b* F 5`CGTGGACATGGCTTTACCTT 3`, *klf2b* R 5`ATGGGAGCTTTTGGTGTACG 3`, *klf4a* F 5`TTGATAGCATGGCACTGAGC 3`, *klf4a* R 5`CCTGCGGAAATCCAGAATAA 3`, *biklf/klf4b/klf17* F 5`ACCCCGGACATGAATTATCA 3`, *biklf/klf4b/klf17* R 5`TGTCCGGTGTGTTTCCTGTA 3`. T3 promoter sequence 5`CATTAACCCTCACTAAAGGGAA 3`was added to 5`end of each of the F primers and T7 promoter sequence 5`TAATACGACTCACTATAGGG 3`was added to 5`end of each of the R primers. RT-PCR and subsequent PCR amplification of corresponding cDNA was performed in a single step using Superscript III One-Step RT-PCR kit (INVITROGEN) following the manufacturer`s instructions. Antisense riboprobes were synthesized using T7 RNA polymerase (ROCHE). The remaining riboprobes used in this study have been kindly donated by other research groups: *dll4* [40] *runx1* [41] and *cmyb* [42].

### Microscopy

Visual assessment of embryos, monitoring of WISH staining and heart rate counting were performed using a Leica S6E stereo microscope (LEICA MICROSYSTEMS)**.** Images of live embryos and WISH images were taken using a Leica M165 FC fluorescent stereo microscope with Leica DFC310 FX camera (both by LEICA MICROSYSTEMS). Fluorescence microscopy was performed using UltraVIEW Vox spinning disc confocal microscope and Volocity v5.3.2 imaging software (both by PERKIN ELMER). Zebrafish embryos were anaesthetised with Tricaine and mounted on a cover slip in 1% low-melting point agarose prior to imaging. Images were taken in 2μm slices across the region of interest and digitally processed by focal plane merging (Z stacking). Merged images were further analysed using ImageJ.

### Western Blotting

Total protein was extracted from genotyped zebrafish embryos at 5dpf (4 embryos per group) using RIPA lysis buffer (SIGMA-ALDRICH) with added Halt Protease and Phosphatase inhibitor cocktail (THERMO SCIENTIFIC). Embryos were homogenised using an Eppendorf pestle (SIGMA-ALDRICH) and left on ice for 30min. Lysate was centrifuged at 14000rpm for 10min at 4°C and the supernatant was separated and protein amounts quantified using a Bradford assay. Protein samples were then mixed with 1M dithiothreitol (DTT) (SIGMA-ALDRICH) and NuPAGE LDS Sample Buffer (LIFE TECHNOLOGIES) heated to 95°C for 5min and loaded onto a NuPAGE NOVEX 4–12% Bis-Tris gel (LIFE TECHNOLOGIES). After the electrophoretic separation, proteins were transferred to Immobilon-P PVDF transfer membranes (MILLIPORE) using the Xcell II Blot Module (LIFE TECHNOLOGIES). After blocking with 5% non-fat milk for 1 hour at RT, transfer membranes were incubated with rabbit anti-mouse polyclonal Klf2 antibody (raised against a polypeptide containing AAs 70–270 of mouse wildtype Klf2a protein) (1:500) (MILLIPORE, Cat.# 09–820) or with a rabbit anti-human β-actin antibody (1:10000) (CELL SIGNALLING, Cat.# 4967) overnight. Membranes were washed in TBSTw (Tris buffered saline plus Tween 20: 136.8mM NaCl, 24.8mM Tris, 0.1% Tween 20 (vol/vol) (all from SIGMA), pH 7.6) for 3x5min on a rocker and incubated with a secondary antibody (goat anti-rabbit polyclonal antibody with conjugated horseradish peroxidase (HRP), 1:1000) (DAKO) for 45min on a rocker. After thorough washing of the membranes, immobilised proteins were detected by the EZ-ECL Chemiluminiscence Detection Kit (BIOLOGICAL INDUSTRIES).

### *klf2a* TALEN Mutagenesis

We followed a published protocol for targeted TALEN mutagenesis [23]. *klf2a* genomic sequence was submitted to the Old TALEN Targeter software at https://tale-nt.cac.cornell.edu/node/add/talen-old. A target site at the 5`end of exon 2 5`TCAACCCATCACCACCTCCACCG/AT[ACACCACCAGCCTACTGGC]AGAGCTTCTGCAGTCTG 3`(19bp spacer sequence between target sequences for L and R TALEN subunits is annotated with square brackets) containing the XcmI (NEB) restriction enzyme site 5`CCANNNNNNNNNTGG 3`was chosen for the mutagenesis. Primers flanking the target site were designed amplifying a 281bp PCR product; *klf2a* TAL XcmI F 5`CAGGCGACTACAGAATGCAA 3`and *klf2a* TAL XcmI R 5`GCCCTCTTGTTTGACTTTGG 3`. Genomic DNA extraction was performed using REDExtract-N-Amp™ Tissue PCR Kit (SIGMA-ALDRICH). XcmI (NEB) restriction enzyme digest (3 hours, 37°C) results in generation of 180bp and 101bp fragments. Successful mutagenesis is indicated by the loss of the restriction site and the presence of an undigested 281bp band after the XcmI (NEB) digest. Following the assembly and capped mRNA synthesis, 1.5ng of capped mRNA coding for the *klf2a* TALEN construct was injected per embryo at one cell stage.

### Statistical Analysis

F1 *klf2a* mutant studies were blinded to whether embryos were homozygous mutants, heterozygotes or wildtype (after imaging and quantification embryos were euthanized and genotyped). Statistical analysis was carried out using GraphPad Prism v5.04 software. Quantitative data are presented as mean ± standard error of the mean (SEM). Statistical comparisons between two groups were made by Student’s t-test. Statistical significance is indicated as: ns = non-significant, * = p<0.05, ** = p<0.01, *** = p<0.001, **** = p<0.0001.

## Results

### The Expression of *klf2a* in Developing Zebrafish Embryos

**Fig 1:**
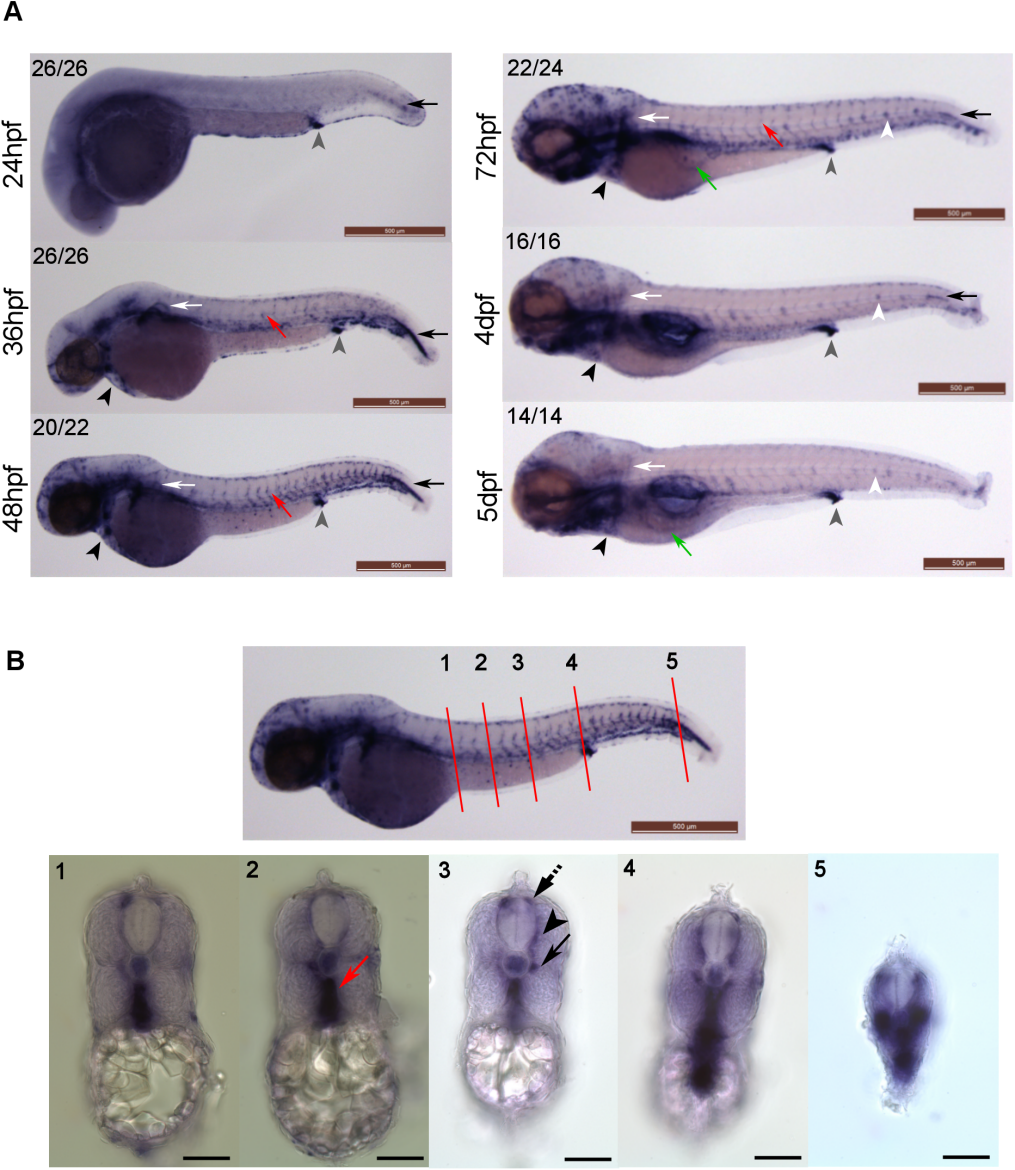
*klf2a* expression patterns in developing zebrafish embryos. **(A)** *klf2a* expression patterns were examined using whole-mount in situ hybridisation. Grey arrowheads indicate cloaca, black arrows indicate cells lateral to the most posterior notochord, white arrows indicate pectoral fin, red arrows indicate trunk vasculature, black arrowheads indicate the cardiac outflow tract, white arrowheads indicate neuromasts and green arrows indicate subintestinal veins (3dpf) or hepatic portal vein (5dpf). Numbers in the top left corners indicate number of embryos with similar staining patterns out of total number of embryos examined. Scale bar = 500μm. **(B)** Cross sections of a 48hpf wildtype embryo showing *klf2a* expression in dorsal aorta (red arrow), parachordal vessel (black arrow), intersegmental vessel (ISV) (black arrowhead) and dorsal longitudinal anastomotic vessel (DLAV) (dotted black arrow). Anatomical positions of sections are indicated by the red lines and numbers on the top panel figure with corresponding cross sections images in the bottom panel. Scale bar = 500μm (top panel) and 100μm (bottom panel).

We analysed *klf2a* expression during zebrafish embryonic development daily from 1 to 5 days post fertilisation (dpf) in wildtype embryos using WISH (Fig 1A). Prior to onset of blood flow (24-26hpf) we could detect only non-vascular *klf2a* expression notably in the cloaca (Fig 1A, grey arrowheads) and in cells abutting the most posterior notochord (Fig 1A, black arrows). After onset of blood flow, *klf2a* was expressed in the developing vasculature, in particular at 48hpf (Fig 1A, red arrow). Cross sections at 48hpf confirmed vascular expression of *klf2a* (Fig 1B).These observations confirm previously published data on *klf2a* expression in early embryonic stages [17]. We next extended our studies to later stages of development to 5dpf and found *klf2a* expression in the trunk vasculature, intersegmental vessels and cardiac outflow tract (Fig 1A, red arrows and black arrowheads respectively). *klf2a* mRNA in the cardiac outflow tract was detectable up to 5dpf, whereas *klf2a* expression in the trunk vasculature and intersegmental vessels was only detectable up to 72hpf (Fig 1A). From 72hpf onwards, *klf2a* was expressed in the subintestinal veins that subsequently form the hepatic vein at 5dpf (Fig 1A, green arrows). We also detected *klf2a* expression in the neuromasts of the lateral line from 72hpf onwards (Fig 1A, white arrowheads).

We confirmed that *klf2a* expression in the zebrafish vasculature is dependent on blood flow using three distinct approaches. These were by preventing cardiac contraction by morpholino antisense knockdown of cardiac troponin T2; preventing aortic blood flow to the trunk due to the aortic occlusion caused by homozygous mutation in *hey2/gridlock*; and finally by chemically-induced cessation of established blood flow between 46-65hpf by incubation in tricaine. When we examined vascular *klf2a* expression, this was reduced in all three models of hindered flow, in keeping with previous studies using *tnnt2* knockdown [3] (S1 Fig).

### Generation of a Stable *klf2a* Mutant Line

**Fig 2:**
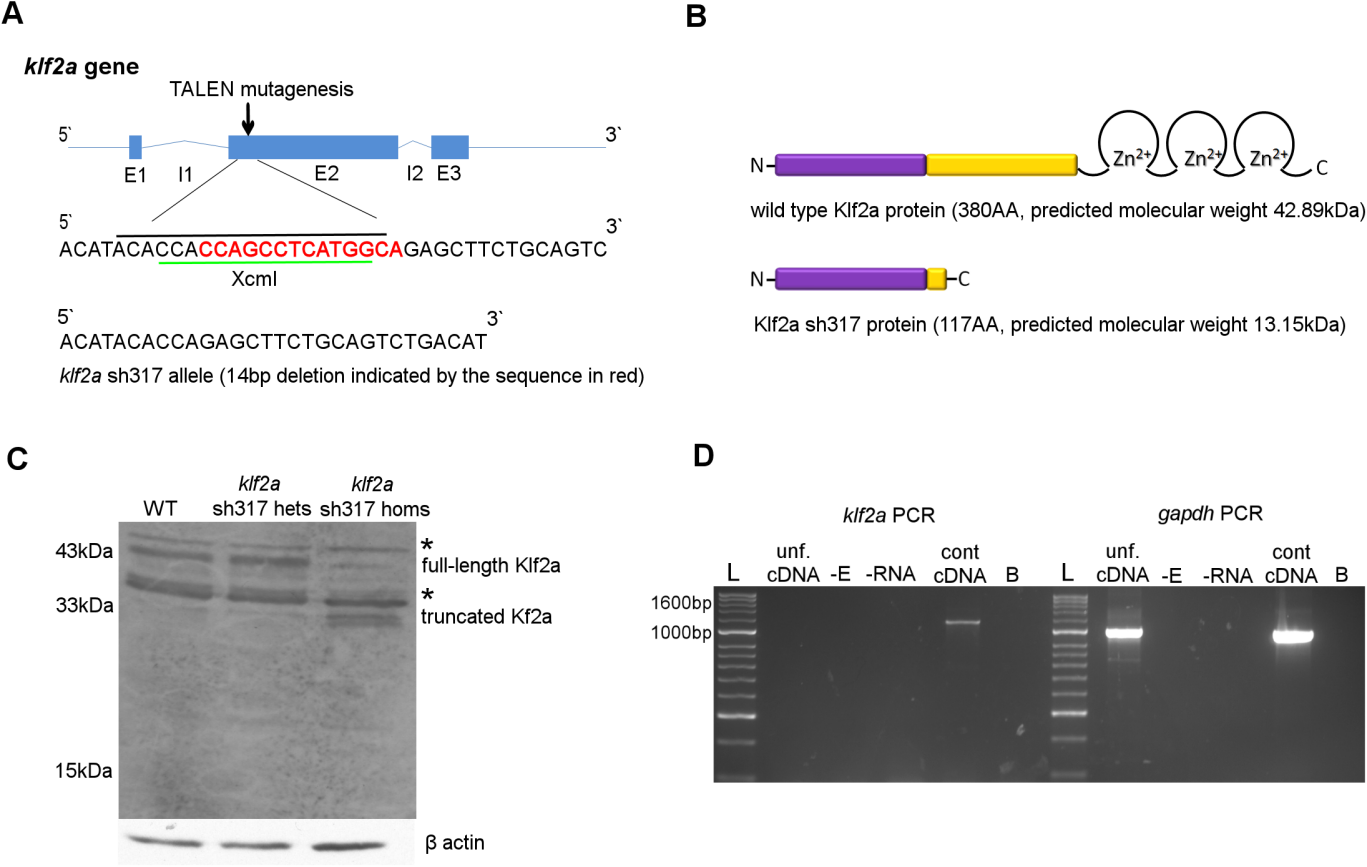
Generation of a stable *klf2a* ^sh317^ mutant line. **(A)** Schematic structure of wildtype *klf2a* gene with genomic sequence at the TALEN mutagenesis site. XcmI restriction site is indicated by the green line and the 19bp spacer for the TALEN structure of choice is indicated by the black line. The 14bp deletion identified in the *klf2a* ^sh317^ allele is indicated by the sequence in red. **(B)** Schematic structure of Klf2a wildtype and Klf2a sh317 mutant proteins with amino acid (AA) lengths and predicted molecular weights in kilodaltons (kDa) Klf2a transactivation domain is in purple and transrepression domain is in yellow colour. **(C)** Substantial reduction of the 43kDa band representing the full-length Klf2a protein can be seen in a western blot of whole-embryo protein samples extracted from the F3 generation of *klf2a* ^sh317^ mutant embryos at 5dpf, compared to wildtype embryos and heterozygous *klf2a* ^sh317^ carriers. Additionally, two bands running at approximately 33kDa can be seen in the *klf2a* ^sh317^ mutant, possibly representing truncated Klf2a. Asterixes indicate bands of approx. molecular weights of 38kDa and 47kDa consistent with expression of Klf4a and Biklf/Klf4b/Klf17 respectively, present in all three lanes in equal intensity. β actin was used as a loading control. **(D)** *klf2a* coding sequence was amplified from the control cDNA originating from 48hpf wildtype embryos giving an expected 1155bp PCR product. No band was detected in the sample with cDNA from unfertilised embryos (unf. cDNA) indicating the absence of maternal *klf2a* mRNA in unfertilised embryos. No 1674bp band was detected in unfertilised cDNA or control cDNA confirming absence of genomic DNA contamination. *gapdh* was used as a positive control. A band of expected size was detected in both the unfertilised eggs cDNA and control cDNA lanes indicating maternal *gapdh* mRNA in unfertilised zebrafish eggs. Abbreviations: -E: negative control with no reverse transcriptase added during reverse transcription (RT). -RNA: negative control with no RNA added during RT. B: blank, no template added to the PCR reaction. Abbreviations: L: Hyperladder II (NEB).

To examine the role of *klf2a* on cardiovascular and haematopoietic development we generated a *klf2a* mutant zebrafish using TALEN. The site for mutagenesis was chosen at the 5`end of exon 2 (Fig 2A). Mutations that produce a premature stop codon in this region are predicted to result in translation of a truncated Klf2a protein lacking the 3 tandem zinc fingers DNA binding motif located at the C terminus of the wildtype Klf2a protein (Fig 2B), rendering it unlikely to function as a transcription factor. We raised TALEN injected embryos and sequenced their genomic DNA and identified a founder carrying an allele with a 14 base pair (bp) deletion that disrupted the reading frame at the target site and generated a premature stop codon 14bp downstream. This allele was named *klf2a* ^sh317^ and is predicted to generate a truncated Klf2a protein with molecular weight of 13.15 kilodalton (kDa) (Fig 2B). All mutant fish from the F1, F2 and F3 generation used in subsequent experiments were genotyped and the *klf2a* ^sh317^ mutation confirmed by sequencing.

To determine the effect of the *klf2a* ^sh317^ mutation on the mRNA transcript, we extracted whole embryonic RNA from 30 pooled embryos from the F3 generation of *klf2a* ^sh317^ mutants. *klf2a* coding sequence was reversely transcribed, PCR amplified, cloned into an expression plasmid and transformed into bacterial cells. 23 separate colonies were sequenced. All confirmed the expected *klf2a* ^sh317^ sequence without any additional transcripts (S2 Fig). We did not therefore find any evidence that the *klf2a* ^sh317^ mutation induces alternative splicing.

Targeted mutation can induce gene loss-of-function by a number of mechanisms. As well as production of a truncated or non-functional protein, the mRNA can be targeted for nonsense mediated decay (NMD) [43]. We examined wither the *klf2a* ^sh317^ mutation induced NMD by performing in situ hybridisation for *klf2a* in wildtypes and homozygous *klf2a* ^sh317^ mutants, but found no difference in mRNA expression (S3 Fig). This, together with the fact that we could amplify and clone the expected mRNA transcript by rt-PCR (S2 Fig) strongly suggests that the *klf2a* ^sh317^ mutation does not induce nonsense-mediated decay.

We next attempted to examine the effect of the *klf2a* ^sh317^ mutation on Klf2a protein expression and size by western blotting in wildtype and *klf2a* ^sh317^ mutant embryos (Fig 2C). Although there are no monoclonal antibodies available against zebrafish Klf2a we were able to obtain and use an anti-mouse polyclonal Klf2a antibody previously shown to bind to zebrafish Klf2a [19]. As might be expected from a polyclonal antibody raised against a largely conserved region of several *Klf* genes this blot shows multiple bands consistent with the presence of Klf2a, in addition to other bands which are consistent with Klf4a and Biklf/Klf4b/Klf17 expression (predicted molecular weights 38kDa and 47kDa respectively; indicated by asterixes in Fig 2C). However, a band likely to represent wildtype Klf2a protein with the correct size of 43kDa was detected in either wildtype or heterozygous *klf2a* ^sh317^ embryos (Fig 2C). This band was greatly reduced in homozygous *klf2a* ^*sh31*^ mutants, with the residual band migrating with a similar molecular weight likely to reflect the expression of Klf2b (predicted molecular weight 41.2kDa). Additionally, two bands of approximately 31-33kDa were detected in *klf2a* ^sh317^ homozygous mutants, probably representing truncated Klf2a protein (Fig 2C). Although larger that the predicted molecular mass of Klf2a sh317 protein, the truncated protein would be expected to be very acidic (ph ~4, versus ~7.8 for full-length protein) and contain a significant proportion of proline residues, both features that contribute to aberrant behaviour on SDS-PAGE.

To determine the presence and potential contribution of maternal *klf2a* mRNA we analysed *klf2a* expression by RT-PCR in unfertilised zebrafish embryos. We found no evidence of maternal *klf2a* mRNA in unfertilised zebrafish eggs (Fig 2D).

Taken together, these data strongly suggest that the *klf2a* ^sh317^ mutation induces a significant truncation of the klf2a protein that is highly likely to perturb its function as a transcription factor.

### Characterisation of the *klf2a* ^sh317^ Mutant Line

**Fig 3:**
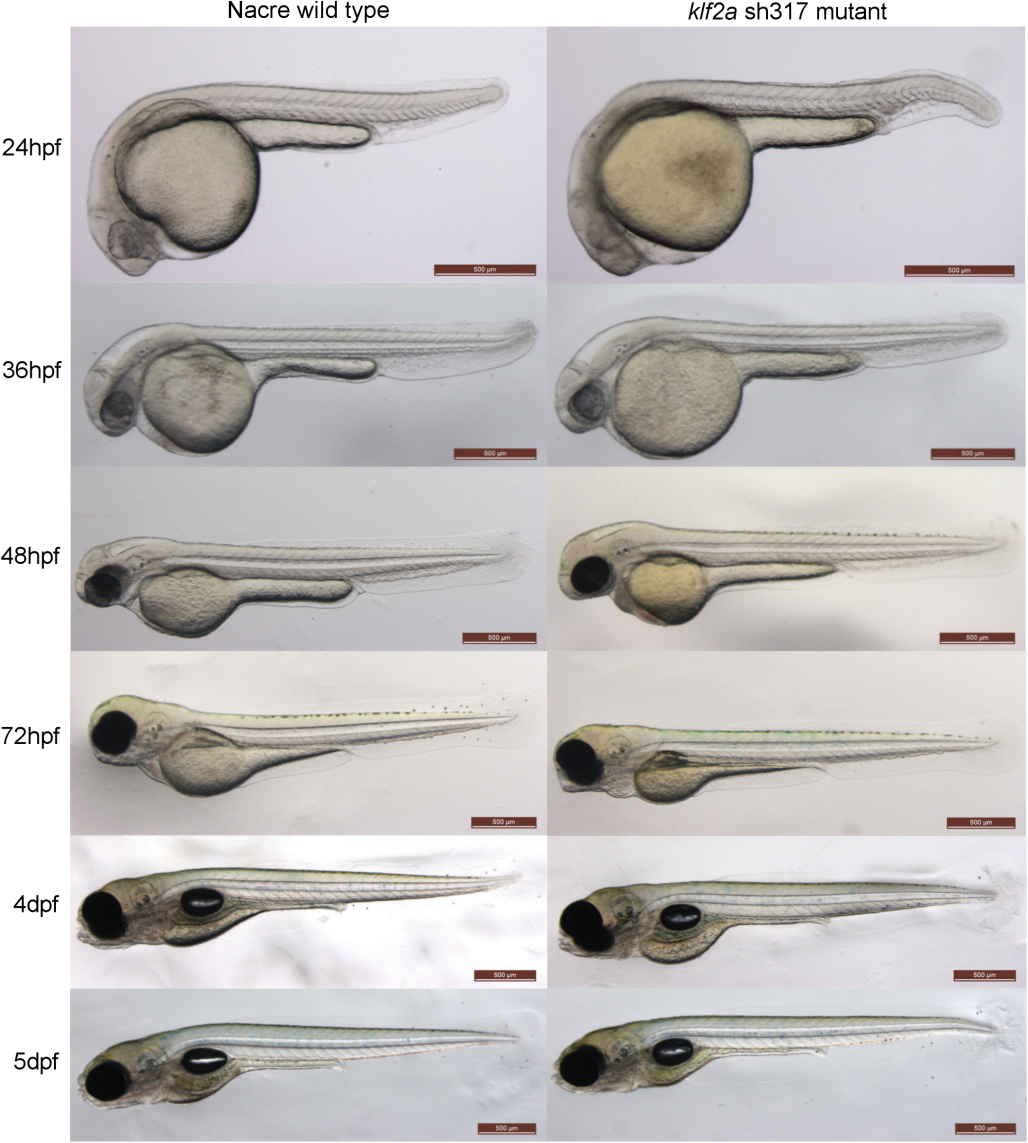
Comparison of the morphology of wildtype and *klf2a* ^sh317^ mutant embryos. There are no obvious morphological differences between wildtype and *klf2a* ^sh317^ mutant embryos up to 5dpf and also beyond up to adulthood (not shown). Scale bar = 500μm.

**Fig 4:**
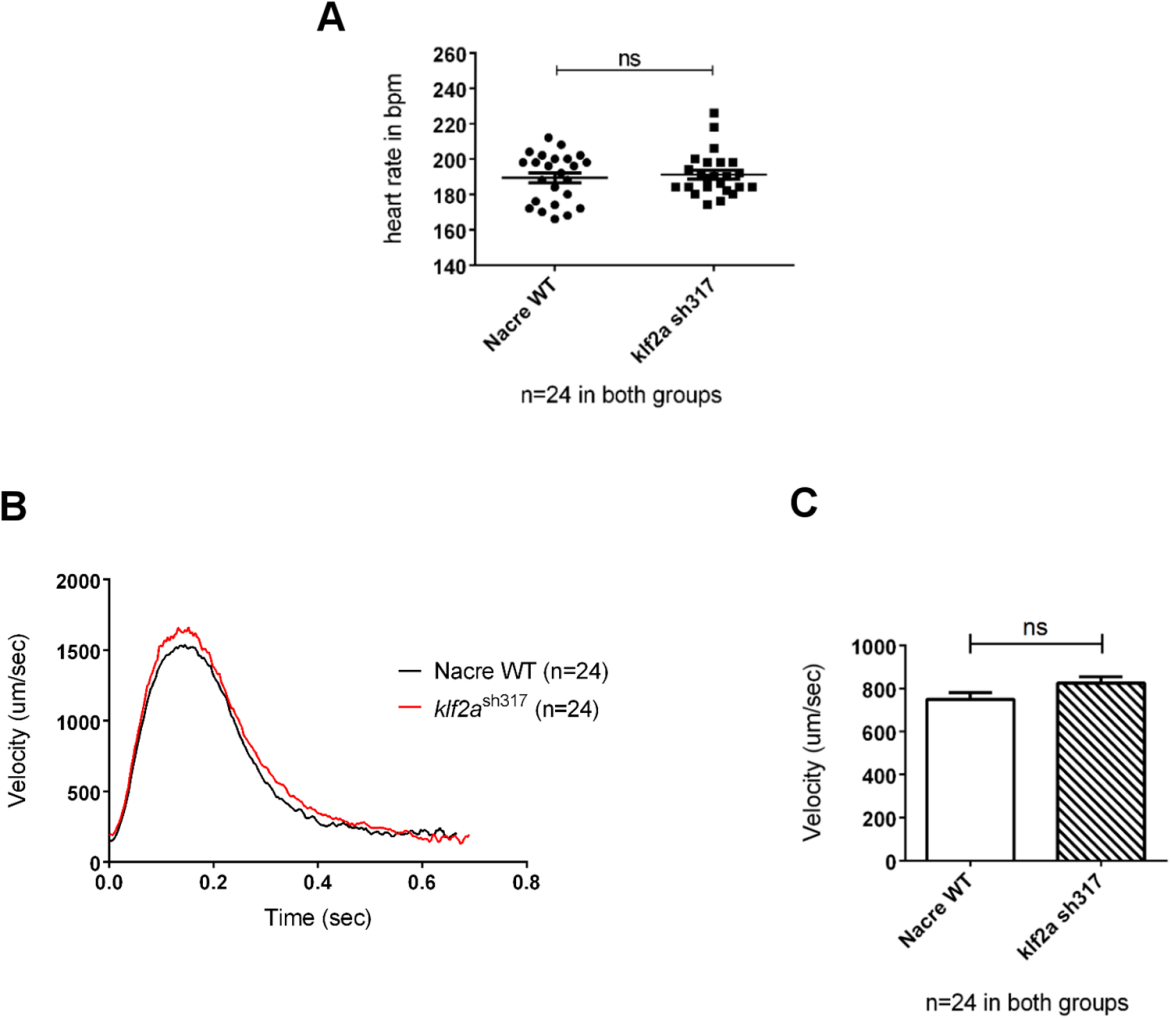
Comparison of heart rates and blood flow velocities of wildtype and *klf2a* ^sh317^ mutant embryos at 48hpf (A) No significant differences in heart rates were observed. (B) Instantaneous blood flow velocities throughout a single cardiac cycle in wildtype and *klf2a* ^sh317^ mutants. (C) Average instantaneous blood flow velocities per cardiac cycle (n = 24, unpaired t-test).

Homozygous *klf2a* ^sh317^ mutants displayed no morphological abnormalities compared to wildtypes by at least 5dpf (Fig 3). In contrast to previously described *klf2a* morphants [15], no pericardial oedema was observed at any stage. *klf2a* ^sh317^ mutants showed no difference in heart rate or blood flow velocity in the dorsal aorta at 48hpf compared to wildtypes, in contrast to the high-output cardiac failure described in *klf2a* morphants [15] (Fig 4). Furthermore, we raised homozygous *klf2a* ^sh317^ mutants to adulthood and both these (F2 generation) and incrossed offspring (F3) were viable and fertile, making late-onset cardiac failure or major cardiovascular defects unlikely and excluding maternal effects that compensate for the mutation when homozygous mutants are derived from heterozygous females.

**Fig 5:**
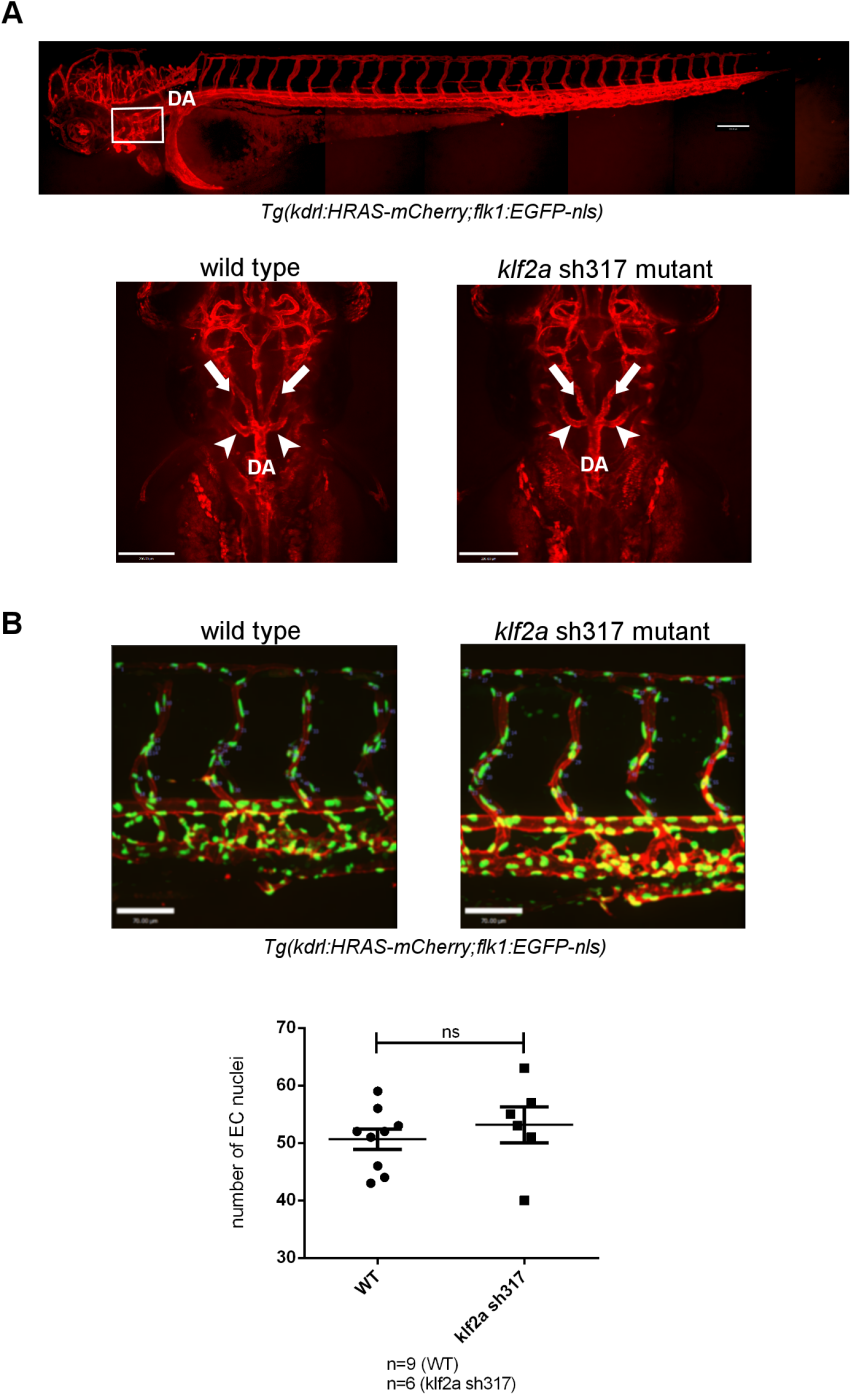
The vascular anatomy of homozygous *klf2a* ^sh317^ mutants. **(A)** Formation of the 5^th^ accessory aortic arch (AA5x) connecting the 5^th^ and 6^th^ aortic arch is not affected in homozygous *klf2a* ^sh317^ mutants. Top panel figure shows vascular anatomy of a zebrafish embryo at 3dpf. White rectangle indicates the location of aortic arches. DA denotes dorsal aorta. Bottom panel figures represent dorsal views on aortic arches. White arrows indicate lateral dorsal aortae. White arrowheads indicate AA5x vessels. Scale bar = 200μm. **(B)** Quantification of endothelial cell nuclei in 4 ISVs closest to the cloaca and corresponding DLAV in WT and *klf2a* ^sh317^ mutants at 3dpf. Scale bar = 70μm.

Next we examined the vascular anatomy of *klf2a* ^sh317^ mutant embryos. We crossed *klf2a* ^sh317^ mutants with the double transgenic line *Tg(kdrl*:*HRAS-mCherry;flk1*:*EGFP-nls*) that differentially labels endothelial cell cytoplasm and nuclei. We found no differences in vascular patterning in *klf2a* ^sh317^ mutants (Fig 5A). Specifically, we examined the trunk vasculature and found no abnormal formation of the axial or intersegmental vessels, nor any difference in endothelial cell number in these regions (Fig 5B and 5C).Formation of the 5^th^ accessory aortic arch (AA5x) has been shown to be impaired either in *klf2a* morphants or in the absence of blood flow [18]. We confirmed that in our hands, formation of the AA5x is blood flow dependent (S4 Fig) but formation of the AA5x was unaffected in *klf2a* ^sh317^ mutants (Fig 5A).

**Fig 6:**
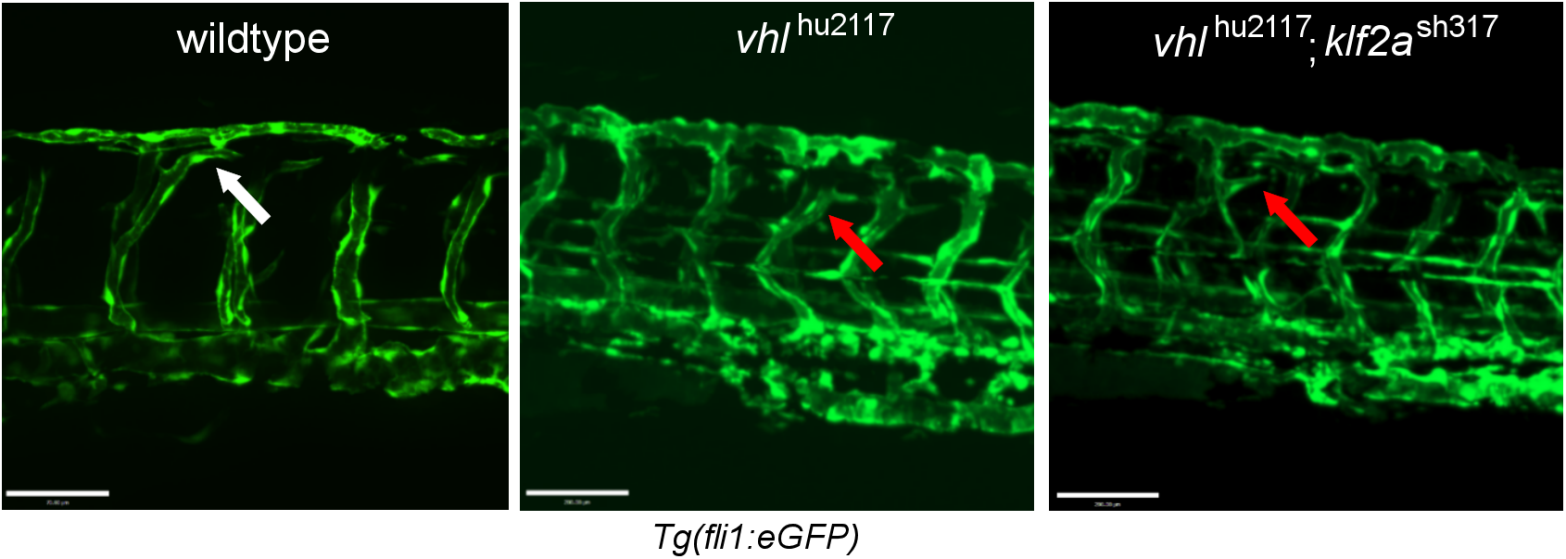
Vascular development in *vhl* ^*hu2117*^ mutants is unaffected by the *klf2a* ^sh317^ mutation. Wildtype embryos exhibit angiogenesis typical for this developmental stage– 3dpf (left figure, white arrow). Homozygous *vhl* ^hu2117^ embryos show enlargement of vessels (ISVs and DLAV) with increased tortuosity and looping of the DLAV (middle figure, right arrow). The same vascular phenotype was be observed in *vhl* ^hu2117^ embryos in a homozygous *klf2a* ^sh317^ background (right figure, red arrow). Confocal images of *Tg*(*fli1*:*eGFP)* zebrafish embryos at 3dpf.. Scale bar = 70μm.

We recently showed that the excessive hypoxic signalling-driven trunk vessel angiogenesis in homozygous *vhl* mutants (*vhl* ^hu2117^) is blood flow dependent [44]. Since *klf2a* is a major endothelial flow responsive transcription factor, we determined the effect of the *klf2a* ^sh317^ mutation on angiogenesis in *vhl* ^hu2117^ mutants. Heterozygotes for *klf2a* ^sh317^ and *vhl* ^hu2117^ in the *Tg(fli1*:*eGFP)* background were incrossed. Embryos homozygous for both *klf2a* ^sh317^ and *vhl* ^hu2117^ still display aberrant intersegmental vessel development (Fig 6) indicating that the *klf2a* ^sh317^ mutation does not alter this process.

**Fig 7:**
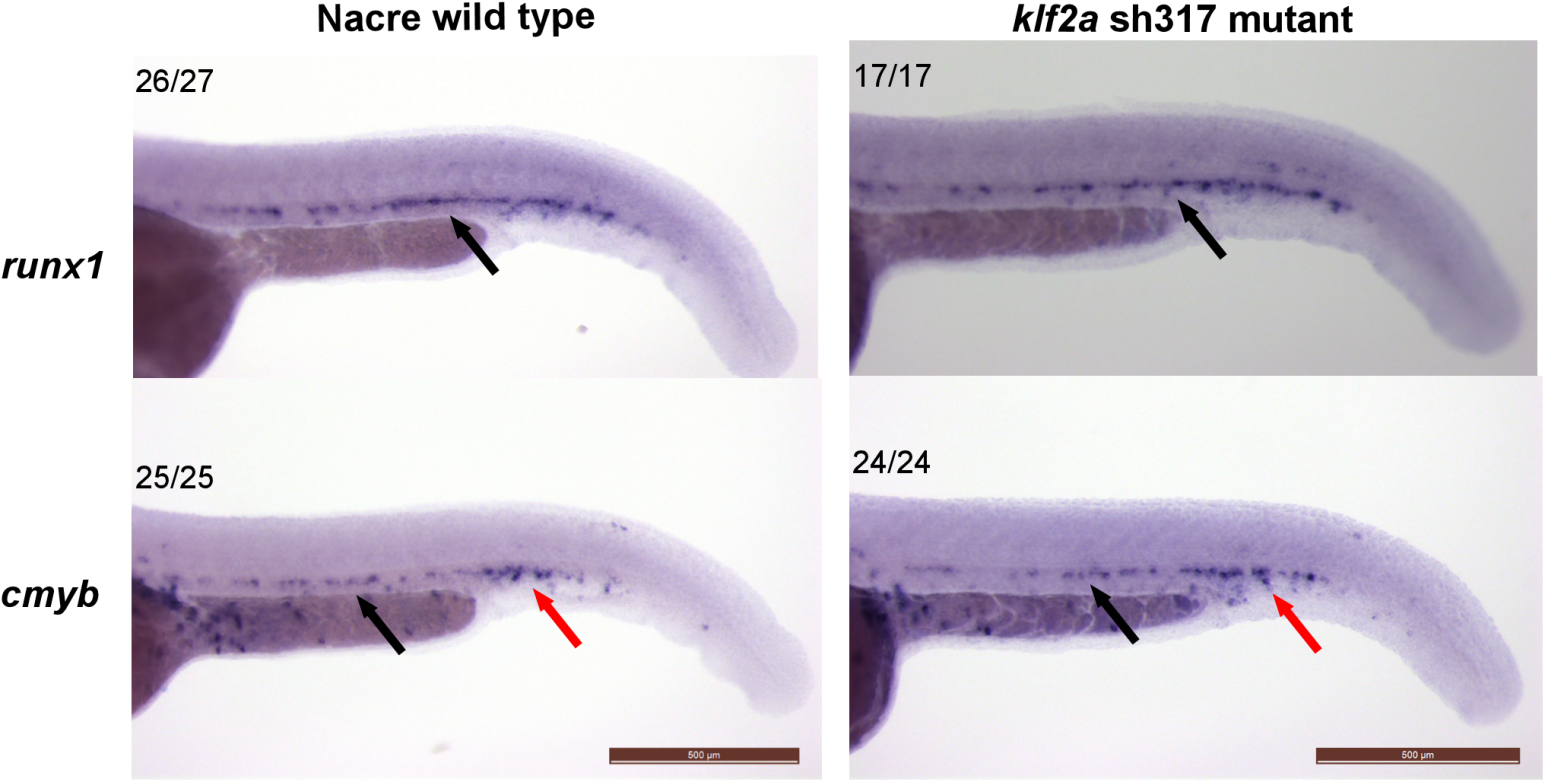
Expression of HSC markers *runx1* and *cmyb* is unaffected in homozygous *klf2a* ^sh317^ mutants at 36hpf. Expression patterns of HSC markers *runx1* (top panel) and *cmyb* (bottom panel) do not differ between the wildtype and *klf2a* ^sh317^ mutants at 36hpf. Black arrows indicate the aorta-gonad-mesonephros (AGM) region and red arrows indicate caudal haematopoietic tissue (CHT). Numbers in the top left corners indicate number of embryos with similar staining patterns out of total number of embryos examined. Scale bar = 500μm.

Since maintenance of haematopoetic stem cell (HSC) gene expression is impaired in *klf2a* morphants, or in the absence of blood flow [19] we examined this in *klf2a* ^sh317^ mutants. While we confirmed that *runx1* and *cmyb* expression was reduced in the absence of blood flow (S5 Fig), expression of both genes was unaffected in homozygous *klf2a* ^sh317^ mutants (Fig 7).

Since homozygous *klf2a* ^sh317^ mutants (unlike *klf2a* morphants) displayed no cardiovascular phenotype, we sought to determine whether this could be explained by redundancy between other klf family members. We examined expression of the three genes phylogenetically closest to *klf2a*, namely the paralog *klf2b* and both *KLF4* orthologs *klf4a* and *biklf/klf4b/klf17*, to assess whether these might be compensating for the *klf2a* ^sh317^ mutation. In addition to the close molecular phylogeny to *KLF2*, *KLF4* has been identified as another endothelial blood flow dependent transcription factor in human and mouse [45, 46]. Vascular expression of *klf2b*, *klf4a* or *biklf/klf4b/klf17* in wildtype zebrafish embryos has not been described [17, 47].

**Fig 8:**
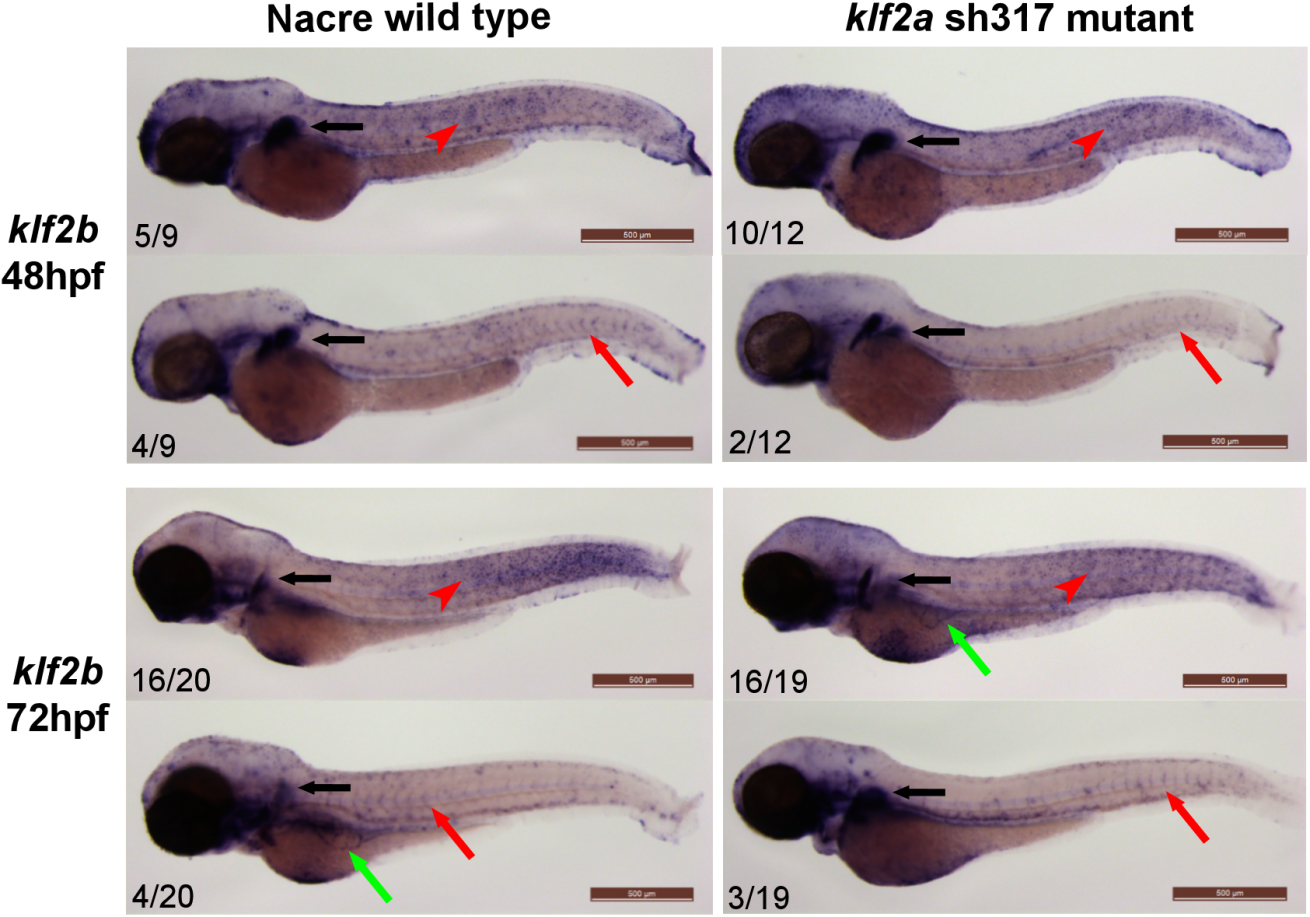
*klf2b* expression patterns do not differ between wildtype and *klf2a* ^sh317^ mutants. *klf2b* mRNA is detectable in the developing pectoral fin bud (black arrows). Most of the embryos examined exhibit *klf2b* mRNA presence on the surface of the embryos representing epidermal cells as described before [17, 48] (red arrowheads). A small proportion of both wildtype and *klf2a* ^sh317^ mutant embryos show vascular staining in the ISVs (red arrows) and/or in the subintestinal veins (green arrows). There were no differences in staining patterns and especially in the level of vascular staining observed between wildtype and *klf2a* ^sh317^ mutant embryos. Figures in bottom left corner of each image indicate the number of embryos with similar staining patterns out of total number of embryos examined. Scale bar = 500μm.

At 48hpf *klf2b* mRNA was detected in the cleithrum and the mesenchyme of the pectoral fin bud (Fig 8, black arrow) and in the epidermal cells (Fig 8, red arrowhead) in keeping with previously published data [17, 48]. Additionally, *klf2b* mRNA was detected in the ISVs of some wildtype and *klf2a* ^sh317^ mutant embryos (Fig 8, red arrows). At 72hpf *klf2b* expression persisted in the pectoral fin and epidermis. As in 48hpf embryos, a small proportion of both wildtype and *klf2a* ^sh317^ mutants at 72hpf exhibited vascular expression of *klf2b* in the ISVs and the subintestinal veins (Fig 8, red arrows). No differences in *klf2b* expression were apparent between wildtype and *klf2a* ^sh317^ mutants.

**Fig 9:**
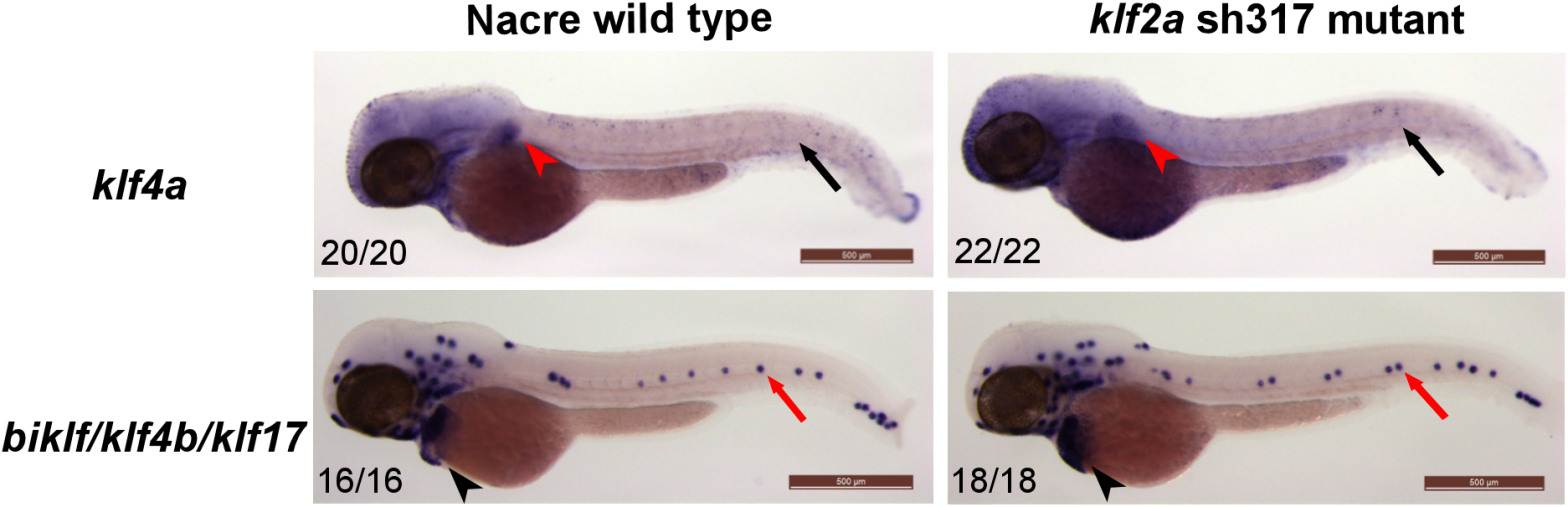
*klf4a and biklf/klf4b/klf17* expression patterns do not differ between wildtype and *klf2a* ^sh317^ mutants at 48hpf. *klf4a* expression was detected in epidermis (black arrows) and pectoral fins (red arrowheads) and *biklf/klf4b/klf17* expression was detected in neuromasts of lateral line organ (red arrows) and in hatching glands (black arrowheads) of wildtype embryos and *klf2a* ^sh317^ mutants in keeping with previously published data [17, 47] No vascular expression of *klf4a* or *biklf/klf4b/klf17* could be detected in any of the wildtype embryos or *klf2a* ^sh317^ mutants examined. Figures in bottom left corner of each image indicate the number of embryos with similar staining patterns out of total number of embryos examined. Scale bar = 500μm.

We detected *klf4a* expression in the epidermis (Fig 9, black arrows) and in the pectoral fin (Fig 9, red arrowheads) of 48hpf wildtype embryos in keeping with previously published data [47]. *klf4a* expression in *klf2a* ^sh317^ mutants was not different to wildtype and no vascular expression of *klf4a* was detected (Fig 9). Similarly, in situ hybridisation for *biklf/klf4b/klf17* at 48hpf confirmed previously published data [17] showing expression in the neuromasts of the lateral line organ (Fig 9, black arrowheads) and in the hatching gland (Fig 9, red arrows). Again, no differences in *biklf/klf4b/klf17* expression were observed between wildytpe and *klf2a* ^sh317^ mutants and no vascular *biklf/klf4b/klf17* expression was detected (Fig 9).

**Fig 10:**
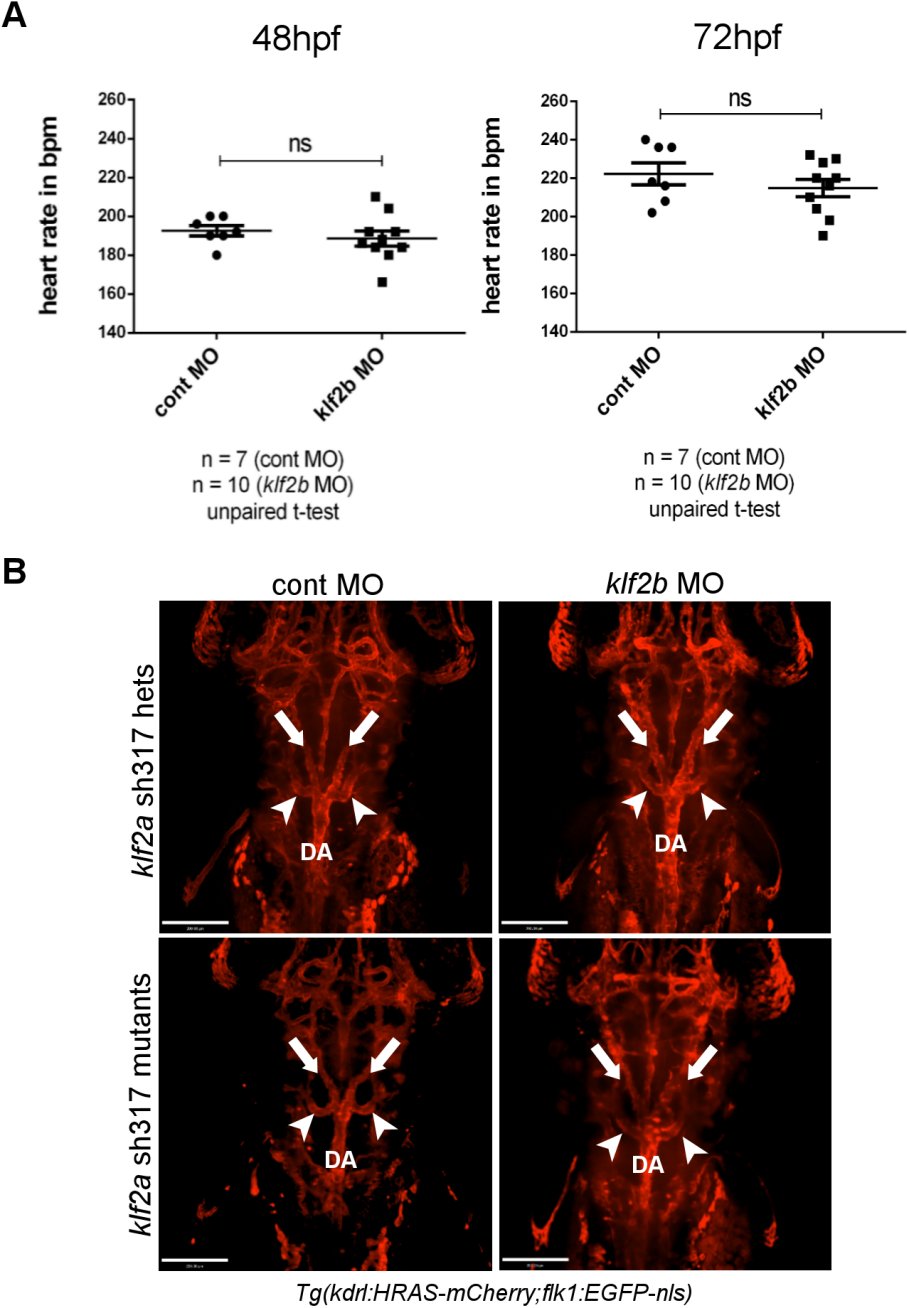
*klf2b* knockdown does not alter heart rate or AA5x formation in *klf2a* ^sh317^ mutants. **(A)** *klf2b* morpholino injection did not affect the heart rate of *klf2a* ^sh317^ mutants at either 48 or 72hpf. **(B)** AA5x vessel formation (white arrowheads) is intact in *klf2b* morphants in either a homozygous or heterozygous *klf2a* ^sh317^ mutant background. White arrows indicate lateral dorsal aortae. DA indicates dorsal aorta. Scale bar = 200μm.

The above detailed experiments found no evidence of compensatory upregulation of *klf2b*, *klf4a*, or *biklf/klf4b/klf17* in *klf2a* ^sh317^ mutants. However, even without evidence of upregulation, normal levels of expression of these genes could conceivable compensate for loss-of-function of *klf2a*. Since only *klf2b* was seen to be expressed in the vasculature, we examined whether morpholino knockdown of *klf2b* induced cardiovascular phenotypes in *klf2a* ^sh317^ mutants. Morpholino-induced *klf2b* knockdown had no effect on either heart rate or AA5x formation in either heterozygous or homozygous *klf2a* ^sh317^ mutants, strongly suggesting that redundancy between *klf2a* and *klf2b* does not explain the lack of a cardiovascular phenotype in *klf2a* ^sh317^ mutants (Fig 10).

**Fig 11:**
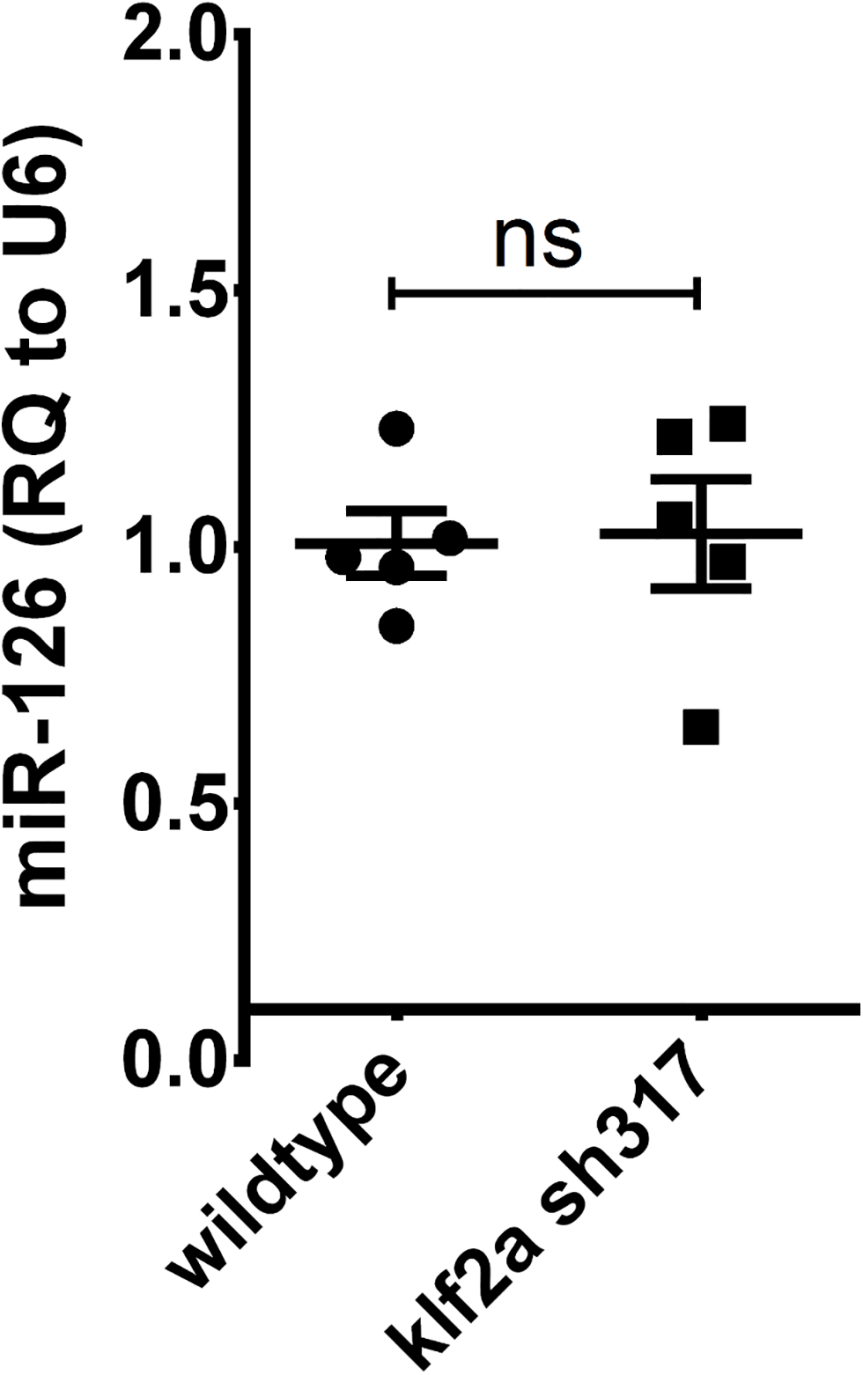
Comparison of miR-126 expression levels in *klf2a* ^sh317^ mutants with wildtypes at 72hpf. Expression levels of miR-126 do not differ between *klf2a* ^sh317^ and wildtypes at the observed time point. Expression of miR-126 was normalized to U6 for relative quantification. Individual points represent relative quantification of miR-126 in a pool of embryos relative to the mean expression in the wild-type group. Summary data is displayed as mean ± SEM.

microRNA 126 (miR-126) has been shown to be upregulated by *klf2a* and morpholino-induced *klf2a* knockdown resulted in reduced miR-126 expression levels in zebrafish [18]. To attempt to further explore the reason for the lack of phenotype in *klf2a* ^sh317^ mutants, we quantified miR-126 levels in 72hpf *klf2a* ^sh317^ mutants compared with wildtypes. We found no reduction in miR-126 levels in *klf2a* ^sh317^ mutants compared to wild types (Fig 11). This suggests that although the mutation clearly induces a significant alteration in the *klf2a* transcript in association with a truncated Klf2a protein, the downstream functions of *klf2a* appear not to be perturbed.

## Discussion

In this study we describe for the first time the generation and characterisation of a stable *klf2a* mutant zebrafish. We find that, in contrast to several studies describing a range of cardiovascular phenotypes in *klf2a* morphant zebrafish, homozygous *klf2a* ^sh317^ mutant embryos display no discernible cardiovascular abnormalities, survive into adulthood and are fertile. There are several possible explanations for the discrepancy between the morphant and *klf2a* ^sh317^ mutant phenotype, a phenomenon that is increasingly being recognised as targeted mutants are generated for genes previously studied using morpholinos [25].

It is possible that some or all of the published *klf2a* morpholino studies are describing off-target or toxic effects of the reagent used, rather than specific effects of *klf2a* knockdown. This is perhaps particularly possible in the case of *klf2a*, expression of which is regulated by blood flow. Non-specific effects that reduce cardiac output would indirectly reduce *klf2a* expression and confound interpretation of the phenotype. This could explain the findings that *klf2a* morpholino knockdown impairs AA5x [18] and HSC formation [19], both of which are impaired in the absence of blood flow. It is harder to ascribe the phenotype of high-output heart failure [15] to off-target effects or toxicity since this is a phenotype not commonly seen in morpholino studies, although the pericardial oedema also reported in this study is commonly induced by high-dose morpholino injections. However, high-output heart failure was not reported in the later *klf2a* morpholino studies, suggesting that it was not observed by these groups.

Conversely, it is possible that the *klf2a* ^sh317^ mutation does not induce loss-of-function and this accounts for the lack of a mutant phenotype. Despite concerns about the reliability of morpholinos, and the increasing preference for using stable mutants, it remains challenging to confirm that a targeted mutation truly induces loss-of-function. The deletion in the genomic DNA sequence of *klf2a* ^sh317^ mutants leads to a frame shift resulting in a premature stop codon that would remove the DNA binding motifs necessary for *klf2a* to function as a transcription factor. Sequencing the cDNA from *klf2a* ^sh317^ mutants found no evidence that the deletion leads to an alternatively spliced transcript that could remain functional. We were fortunate to obtain an antibody with at least some degree of cross-reactivity against zebrafish Klf2a, since a lack of suitable antibodies prevents assessment of the effect of targeted mutation on protein expression in the case of most genes. Western blotting with this antibody confirmed loss of wildtype Klf2a in *klf2a* ^sh317^ mutants. However, we did not detect a band of the predicted size of the mutant transcript (13kDa) but instead detected a novel band of around 33kDa. Although it is possible that this represents the predicted mutant protein with additional post-translational modifications, it is also possible that some mechanism has allowed the ribosome to produce a truncated yet functional Klf2a protein in the face of the *klf2a* ^sh317^ mutation.

After initial submission of this paper, Rossi et al. published a description of a compensatory mechanism that rescues the effect of *egfl7* mutants but not of *egfl7* morpholino knockdown, via upregulation of Emilins [49]. Since the *klf2a* ^sh317^ mutation seems highly likely to perturb *klf2a* function, yet does not affect miR-126 levels, we consider it most likely that a similar mechanism exists for *klf2a*. We have shown that this compensation is not mediated by *klf2b*, but it is entirely possible that other genes may rescue the effect of the the *klf2a* ^sh317^ mutation. Since forward mutagenesis screens in zebrafish have been highly successful, not least for cardiovascular phenotypes [50], such mechanisms appear not to compensate for many genes. However, the existence of such mechanisms may explain the observation that in a large-scale zebrafish mutagenesis study only 6% of disruptive mutations in protein-coding genes induced an observable phenotype [27].

In summary, our work shows that even in the face of clear evidence of a potentially disruptive mutation induced in a gene of interest, it is currently very difficult to be certain that this leads to loss-of-function, and hence to be confident about the role of the gene in embryonic development.

## Supporting Information

S1 Checklist NC3Rs ARRIVE Guidelines Checklist. (PDF)

S1 Fig *klf2a* vascular expression is blood flow-dependent. *klf2a* is expressed in zebrafish embryonic vasculature of a WT embryo at 48hpf. Cessation of blood flow in the trunk vasculature by an occlusion of proximal aorta in the *gridlock* mutants (indicated by a green arrow) results in a complete loss of *klf2a* vascular expression distally to the occlusion. Blockage of embryonic heart contractions by *tnnt2* MO results in significantly decreased *klf2a* vascular expression. Pharmacological inhibition of heart contractions by tricaine from 32 to 48hpf results in significantly decreased *klf2a* vascular expression. Interestingly tricaine also reduces *klf2a* expression in the heart region and in the cells lateral to the most posterior notochord. Red arrows indicate trunk vasculature, black arrows indicate the cells lateral to most posterior notochord, white arrows indicate pectoral fins, black arrowheads indicate the cardiac outflow tract and white arrowheads indicate cloaca. Numbers in top right corners indicate the number of embryos with similar staining pattern out of all embryos examined. *klf2a* riboprobe used. Scale bar = 500μm.
(TIF)

S2 Fig *klf2a* wildtype and *klf2a* ^sh317^ coding sequences, primary protein structures and *klf2a* ^sh317^ cDNA sequencing. **(A)** *klf2a* wildtype and *klf2a* ^sh317^ coding sequences. Exons are colour coded in alternating black and blue colours. Nucleotides within the 19bp spacer between the *klf2a* TALEN L and R subunit binding sites are underlined. Targeted TALEN-induced mutagenesis occurs around this region. Codons coding for amino acids that bind the zinc atoms within the 3 tandem C_2_H_2_ zinc fingers at the C terminus of wildtype Klf2a protein are highlighted in red. 14bp deletion in the *klf2a* ^sh317^ coding sequence causes disruption of the reading frame. **(B)** Primary protein structures of Klf2a WT and Klf2a sh317 proteins are aligned. Cysteine (C) and histidine (H) amino acids that bind zinc atoms within the 3 tandem C_2_H_2_ zinc fingers at the C terminus of wildtype Klf2a protein are highlighted in red. **(C)** Sequencing of reversely transcribed *klf2a* ^sh317^ mRNA confirmed the presence of expected sequence in 23/23 sequencing reactions without any additional transcripts detected. Sequence highlighted in blue (ACACCA) indicates the area of targeted mutagenesis in *klf2a* gene.
(TIF)

S3 Fig *klf2a* expression patterns in *klf2a* ^sh317^ mutants. No differences in *klf2a* WISH staining patterns were detected in *klf2a* ^sh317^ mutant embryos when compared to wildtype counterparts at examined time points indicating the absence of nonsense-mediated mRNA decay (NMD) in *klf2a* ^sh317^ mutants. Grey arrowheads indicate cloaca, black arrows indicate cells lateral to the most posterior notochord, white arrows indicate pectoral fin, red arrows indicate trunk vasculature, black arrowheads indicate the cardiac outflow tract, white arrowheads indicate neuromasts and green arrows indicate subintestinal veins. Numbers in the bottom left corners indicate number of embryos with similar staining patterns out of total number of embryos examined. Scale bar = 500μm.
(TIF)

S4 Fig Formation of the AA5x vessel in blood flow dependent. **(A)** Vascular anatomy of a zebrafish embryo at 3dpf. White rectangle indicates the location of aortic arches. DA denotes dorsal aorta. Scale bar = 200μm. **(B)** Formation of AA5x vessels in unaffected in the WT embryos (untreated controls) as indicated by the white arrowheads. White arrows point at lateral dorsal aortae. AA5x vessel is missing in embryos treated with tricaine (middle panel) and with BDM (right figure) which both prevent blood flow (green arrowheads). Formation of lateral dorsal aortae is unaffected (white arrows). These findings confirm the previously published data [18]. Scale bar = 70μm.
(TIF)

S5 Fig Expression of HSC markers *runx1* and *cmyb* is blood flow dependent. Expression patterns of HSC markers *runx1* (top panel) and *cmyb* (bottom panel) is significantly diminished in the *tnnt2* morphants which lack blood flow confirming previously published data [19]. Black arrows indicate the aorta-gonad-mesonephros (AGM) region and red arrows indicate caudal haematopoietic tissue (CHT). Numbers in the top left corners indicate number of embryos with similar staining patterns out of total number of embryos examined. Scale bar = 500μm.
(TIF)
