# Supplementary figures and images for: klf2a sh317 Mutant Zebrafish Do Not Recapitulate Morpholino-Induced Vascular and Haematopoietic Phenotypes
Source: PLoS One. 2015 Oct 27;10(10):e0141611. doi: 10.1371/journal.pone.0141611 (PMC4624238; doi:10.1371/journal.pone.0141611)

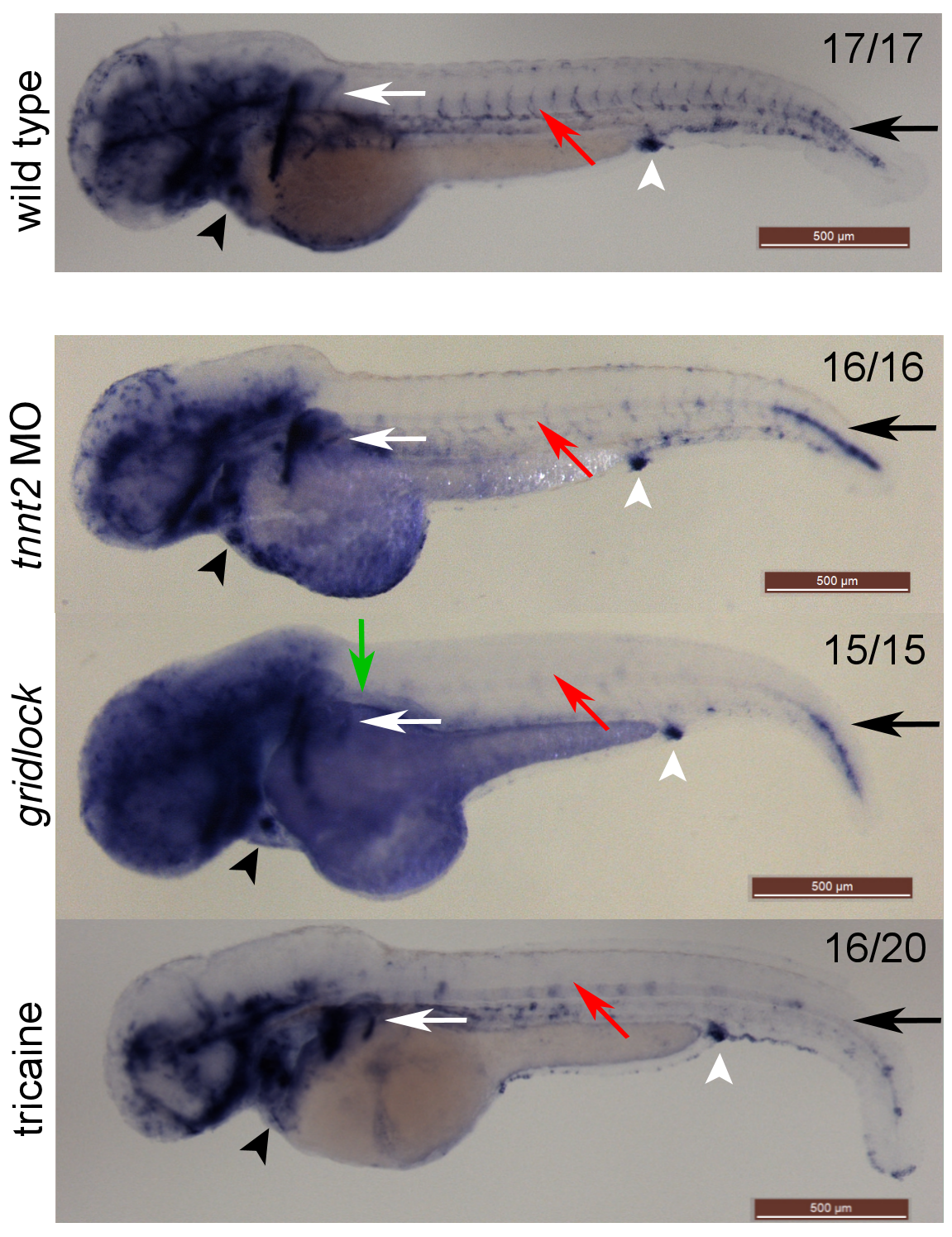

Supplement: S1 Fig — klf2a is expressed in zebrafish embryonic vasculature of a WT embryo at 48hpf. Cessation of blood flow in the trunk vasculature by an occlusion of proximal aorta in the gridlock mutants (indicated by a green arrow) results in a complete loss of klf2a vascular expression distally to the occlusion. Blockage of embryonic heart contractions by tnnt2 MO results in significantly decreased klf2a vascular expression. Pharmacological inhibition of heart contractions by tricaine from 32 to 48hpf results in significantly decreased klf2a vascular expression. Interestingly tricaine also reduces klf2a expression in the heart region and in the cells lateral to the most posterior notochord. Red arrows indicate trunk vasculature, black arrows indicate the cells lateral to most posterior notochord, white arrows indicate pectoral fins, black arrowheads indicate the cardiac outflow tract and white arrowheads indicate cloaca. Numbers in top right corners indicate the number of embryos with similar staining pattern out of all embryos examined. klf2a riboprobe used. Scale bar = 500μm. (TIF) [file pone.0141611.s002.tif]

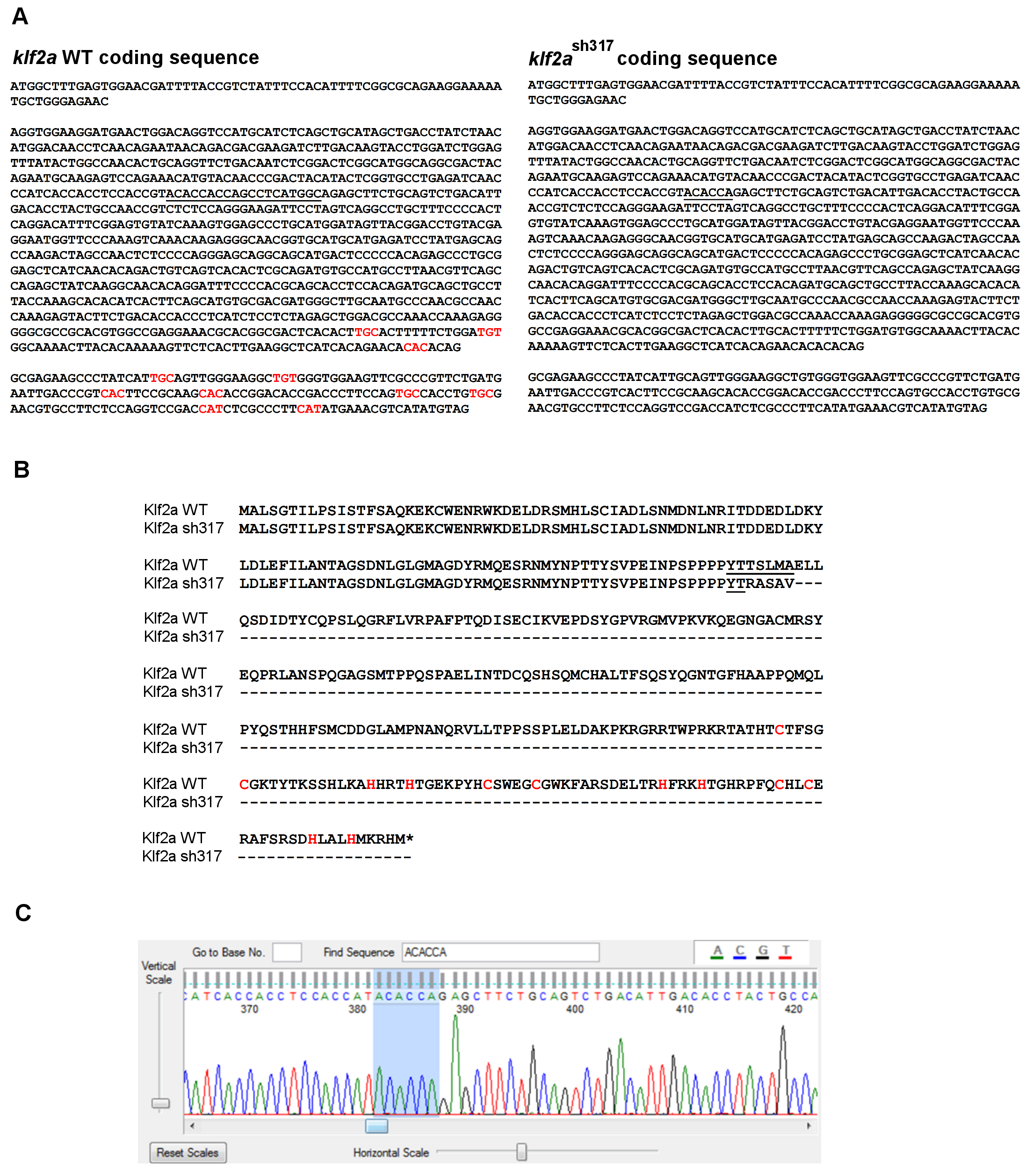

Supplement: S2 Fig — (A) klf2a wildtype and klf2a sh317 coding sequences. Exons are colour coded in alternating black and blue colours. Nucleotides within the 19bp spacer between the klf2a TALEN L and R subunit binding sites are underlined. Targeted TALEN-induced mutagenesis occurs around this region. Codons coding for amino acids that bind the zinc atoms within the 3 tandem C2H2 zinc fingers at the C terminus of wildtype Klf2a protein are highlighted in red. 14bp deletion in the klf2a sh317 coding sequence causes disruption of the reading frame. (B) Primary protein structures of Klf2a WT and Klf2a sh317 proteins are aligned. Cysteine (C) and histidine (H) amino acids that bind zinc atoms within the 3 tandem C2H2 zinc fingers at the C terminus of wildtype Klf2a protein are highlighted in red. (C) Sequencing of reversely transcribed klf2a sh317 mRNA confirmed the presence of expected sequence in 23/23 sequencing reactions without any additional transcripts detected. Sequence highlighted in blue (ACACCA) indicates the area of targeted mutagenesis in klf2a gene. (TIF) [file pone.0141611.s003.tif]

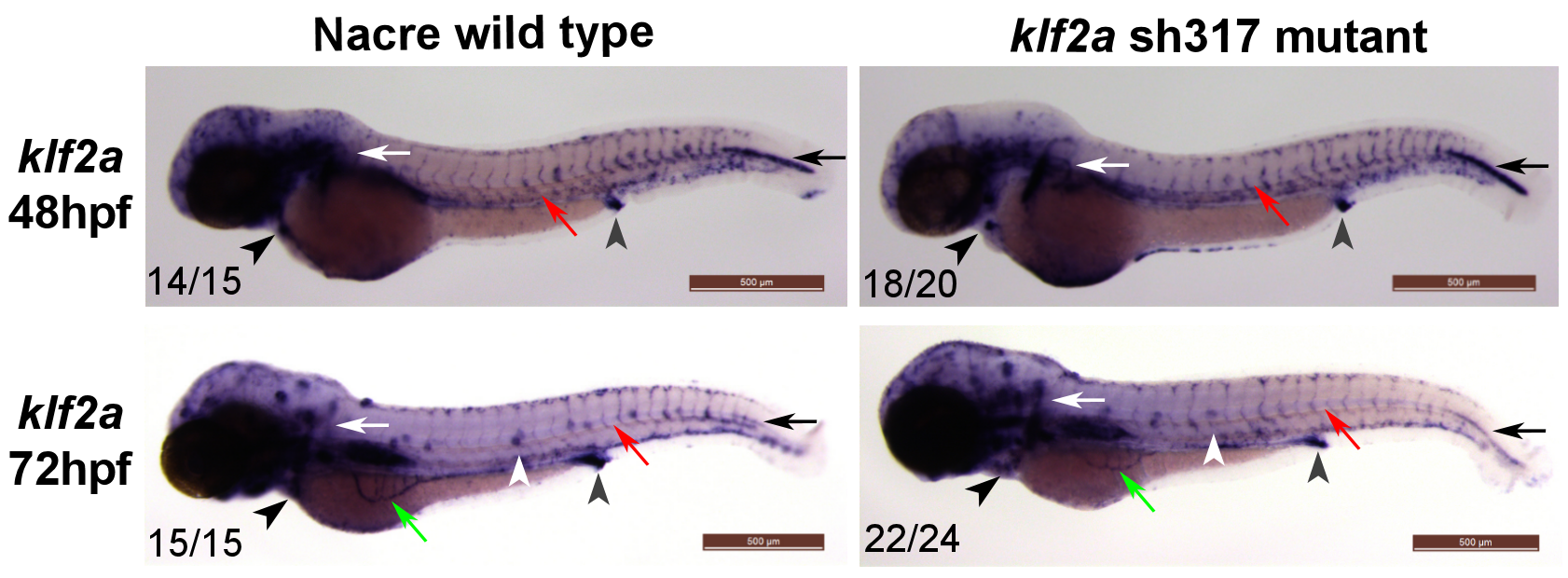

Supplement: S3 Fig — No differences in klf2a WISH staining patterns were detected in klf2a sh317 mutant embryos when compared to wildtype counterparts at examined time points indicating the absence of nonsense-mediated mRNA decay (NMD) in klf2a sh317 mutants. Grey arrowheads indicate cloaca, black arrows indicate cells lateral to the most posterior notochord, white arrows indicate pectoral fin, red arrows indicate trunk vasculature, black arrowheads indicate the cardiac outflow tract, white arrowheads indicate neuromasts and green arrows indicate subintestinal veins. Numbers in the bottom left corners indicate number of embryos with similar staining patterns out of total number of embryos examined. Scale bar = 500μm. (TIF) [file pone.0141611.s004.tif]

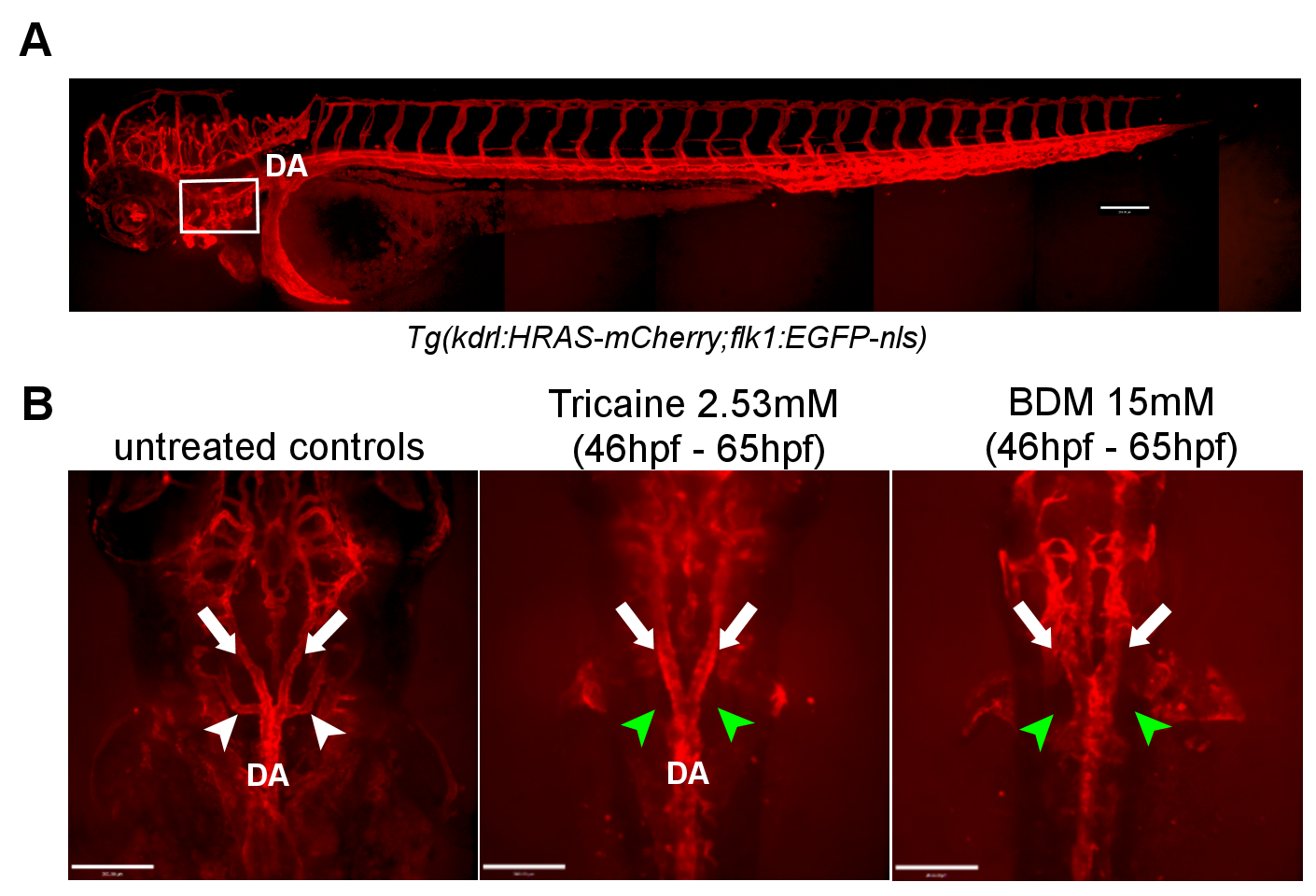

Supplement: S4 Fig — (A) Vascular anatomy of a zebrafish embryo at 3dpf. White rectangle indicates the location of aortic arches. DA denotes dorsal aorta. Scale bar = 200μm. (B) Formation of AA5x vessels in unaffected in the WT embryos (untreated controls) as indicated by the white arrowheads. White arrows point at lateral dorsal aortae. AA5x vessel is missing in embryos treated with tricaine (middle panel) and with BDM (right figure) which both prevent blood flow (green arrowheads). Formation of lateral dorsal aortae is unaffected (white arrows). These findings confirm the previously published data [18]. Scale bar = 70μm. (TIF) [file pone.0141611.s005.tif]

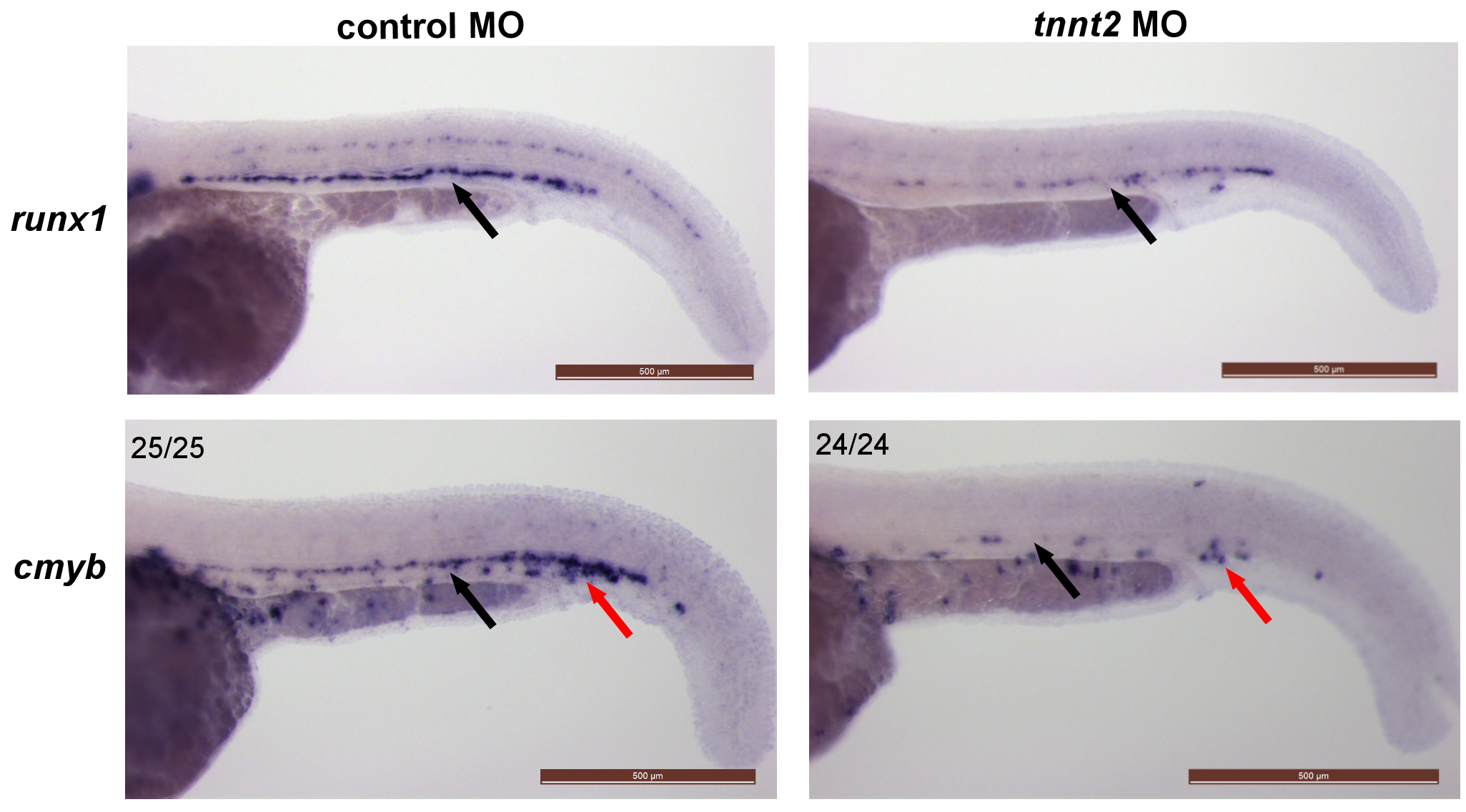

Supplement: S5 Fig — Expression patterns of HSC markers runx1 (top panel) and cmyb (bottom panel) is significantly diminished in the tnnt2 morphants which lack blood flow confirming previously published data [19]. Black arrows indicate the aorta-gonad-mesonephros (AGM) region and red arrows indicate caudal haematopoietic tissue (CHT). Numbers in the top left corners indicate number of embryos with similar staining patterns out of total number of embryos examined. Scale bar = 500μm. (TIF) [file pone.0141611.s006.tif]
